# Accelerated basement membrane remodeling and serum matrix fragments as biomarkers of fibrosis in Alport syndrome

**DOI:** 10.1016/j.celrep.2026.117356

**Published:** 2026-05-15

**Authors:** Rebecca Preston, Anna Hoyle, Emily Williams, Alana Stevenson Harris, Ko Tsutsui, Chloe Williams, Catherine Park, David Spiller, Leo Zeef, Solveig Skovlund Groen, Federica Genovese, Joan Chang, Qing-Jun Meng, Joe Swift, Alexander Eckersley, Rachel Lennon

**Affiliations:** 1 Division of Cell Matrix Biology & Regenerative Medicine, School of Biological Sciences, Faculty of Biology Medicine and Health, The University of Manchester, Manchester Academic Health Science Centre, Manchester M13 9PT, UK; 2 Manchester Cell-Matrix Centre, School of Biological Sciences, Faculty of Biology Medicine and Health, The University of Manchester, Manchester Academic Health Science Centre, Manchester M13 9PT, UK; 3 Kennedy Institute of Rheumatology, The University of Oxford, Oxford OX3 7FY, UK; 4 Bioimaging Core Facility, School of Biological Sciences, Faculty of Biology Medicine and Health, The University of Manchester, Manchester M13 9PT, UK; 5 Bioinformatics Core Facility, School of Biological Sciences, Faculty of Biology Medicine and Health, The University of Manchester, Manchester M13 9PT, UK; 6 Cardiovascular and Renal Research, Nordic Bioscience, Herlev Hovedgade 205, 2730 Herlev, Denmark; 7 Division of Molecular & Cellular Function, School of Biological Sciences, Faculty of Biology Medicine and Health, The University of Manchester, Manchester Academic Health Science Centre, Manchester M13 9PT, UK; 8 Division of Musculoskeletal & Dermatological Sciences, School of Biological Sciences, Faculty of Biology Medicine and Health, The University of Manchester, Manchester Academic Health Science Centre, Manchester M13 9PT, UK; 9 Department of Paediatric Nephrology, Royal Manchester Children’s Hospital, Manchester University Hospitals NHS Foundation Trust, Manchester Academic Health Science Centre, Manchester M13 9WL, UK

## Abstract

Chronic kidney disease (CKD) is characterized by fibrosis. Alport syndrome, a common monogenic form of CKD caused by collagen type IV variants, leads to basement membrane defects and progressive fibrosis. Understanding extracellular matrix dynamics is crucial for identifying disease-specific biomarkers and optimizing therapeutic timing. We applied a discovery approach combining stable ^13^C-lysine metabolic labeling with proteomics, RNA sequencing, super-resolution imaging, and bioinformatics to define temporal and spatial kidney matrix dynamics in Alport mice. Transcriptomic profiling revealed enriched matrix degradation pathways and upregulated proteases, while ^13^C-lysine proteomics demonstrated altered abundance and accelerated turnover of basement membrane components. Super-resolution imaging confirmed matrix protein disorganization, and peptide location fingerprinting mapped damage modifications across ~40 matrix proteins, predicting fragmentation in collagens, laminins, and nidogens. Predicted matrix fragments were detectable in serum from children with Alport variants versus healthy controls, linking basement membrane turnover and fibrosis with clinically accessible biomarkers for CKD and other fibrotic disorders.

## Graphical abstract

**Graphical abstract:**
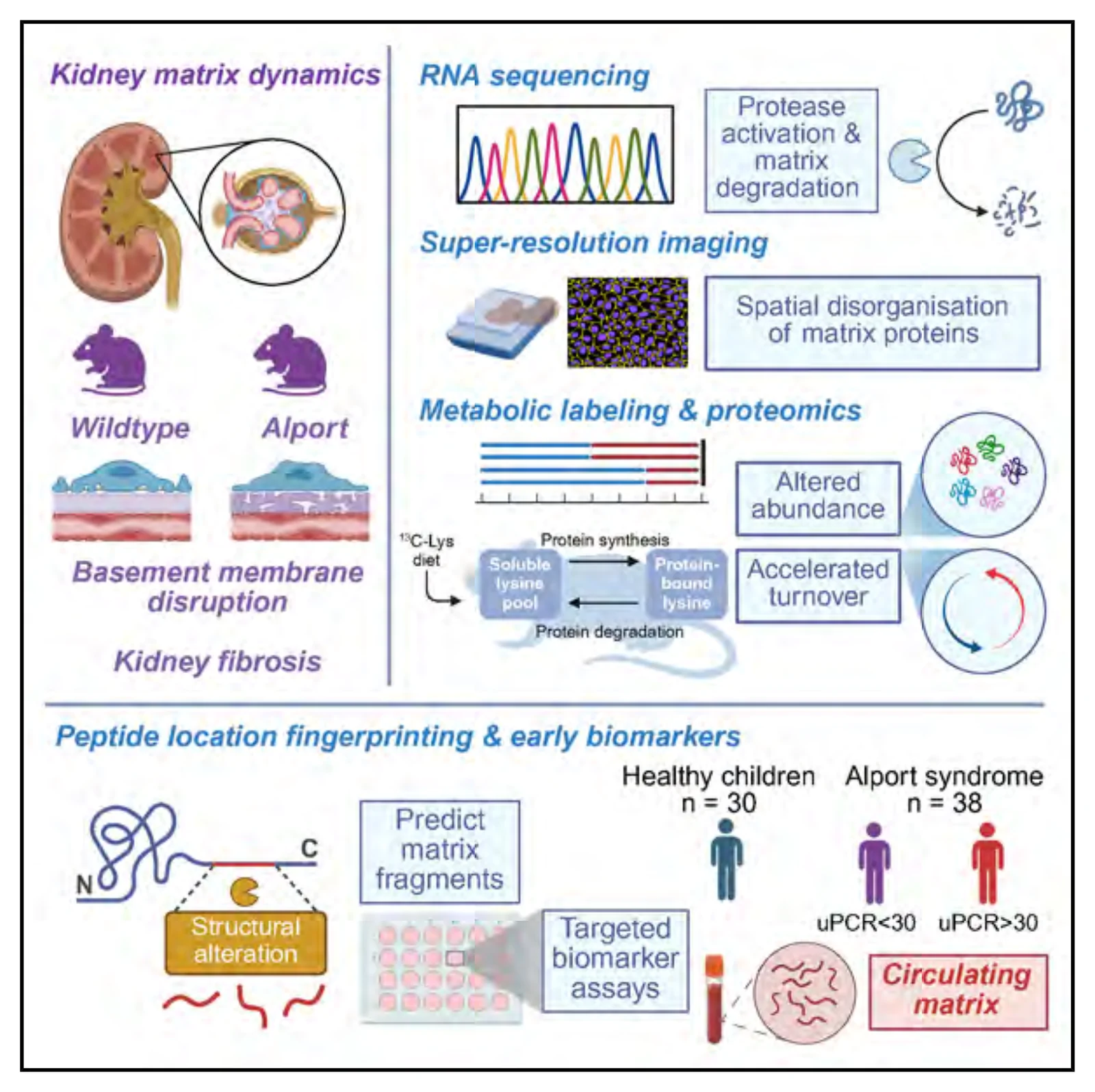

## Introduction

The extracellular matrix is a complex network of macromolecules that provides structural support to tissues and regulates key cellular processes such as proliferation, survival, migration, and differentiation. It is broadly organized into two components: basement membranes, which are dense, sheet-like structures, and the more loosely arranged interstitial matrix.^1^ Basement membranes serve as selective barriers and structural scaffolds in multiple tissues, including the kidney glomerulus, lung alveoli, skin, gut epithelium, placenta, and blood-brain barrier. Matrix proteins, collectively termed the matrisome, can be classified as core matrisome or matrix-associated proteins.^2^ The core matrisome comprises structural components, including collagens, glycoproteins, and proteoglycans,^3,4^ while matrix-associated proteins include matrix regulators, affiliated proteins, and secreted factors. Matrisome proteins are essential for maintaining tissue-specific functions,^4–6^ and pathological changes in these proteins are associated with almost all human diseases.^5–7^ Matrix homeostasis requires tight regulation of synthesis and degradation. When this is perturbed, excessive matrix accumulation can occur, which is the hallmark of fibrosis, a feature implicated in nearly 45% of deaths in the Western world.^8^ Deciphering the mechanisms that lead to abnormal matrix deposition is key to preventing fibrosis and preserving organ function.

In the kidney, basement membranes have a major functional role in the glomerulus. The glomerular basement membrane lies between endothelial cells and podocytes and is essential for selective filtration. The mature glomerular basement membrane is primarily composed of type IV collagen heterotrimers (α3,α4,α5), laminins (mainly α5β2γ1), nidogens, and heparan sulfate proteoglycans such as agrin and perlecan.^9^ Disruption of this specialized matrix occurs in many individuals with chronic kidney disease (CKD), which affects 11%–13% of the global population.^10^ Regardless of the initiating cause, progressive kidney fibrosis is the common histological endpoint of CKD.^11^ Alport syndrome is a common genetic form of CKD caused by variants in *COL4A3, COL4A4*, or *COL4A5* genes, which impair the developmental switch from the fetal (α1,α1,α2) to the adult (α3,α4,α5) collagen IV network.^9^ This leads to a structurally compromised glomerular basement membrane (GBM), proteinuria, and progressive kidney fibrosis. As such, Alport syndrome serves as a valuable model to study basement membrane remodeling and kidney fibrosis.

Over the last decade, proteomic technologies have transformed our understanding of tissue-specific matrix composition and how this is influenced by disease.^12–16^ While traditional proteomic studies provide snapshots of protein abundance, they do not capture the dynamic processes of protein turnover within tissues. Protein turnover, the balance between protein synthesis and degradation, is fundamental to tissue maintenance. Although basement membranes are considered to be stable and rigid scaffolds with components having long half-lives in the order weeks,^17,18^ recent studies have revealed a far more dynamic nature. For example, in invertebrate models such as *C. elegans* and *Drosophila*, live imaging of fluorescently tagged basement membrane components has shown rapid turnover with half-lives as short as 7–10 h.^19,20^ However, it is challenging to simultaneously profile dynamics of multiple proteins with this approach. To address this, metabolic labeling combined with mass spectrometry offers a powerful means to assess protein turnover on a global scale. Use of stable isotope-labeled diets, such as ^15^N or ^13^C lysine, allows quantification of newly synthesized versus pre-existing proteins, and while early ^15^N-based studies estimated global turnover in kidney tissue,^21^ the approach was limited by broad isotope distributions and metabolic recycling,^22^ hindering the precision of half-life calculations. In contrast, ^13^C-lysine labeling provides higher specificity, allowing precise turnover measurements of many more individual proteins based on predictable mass shifts in proteolytic peptides. This strategy has been successfully applied in mammalian tissues including liver, brain, cartilage, and skin^23–27^; however, proteome-wide turnover data for the kidney are not yet available.

Another emerging approach for understanding protein turn-over and degradation is peptide location fingerprinting (PLF), a proteomic analysis tool capable of detecting structural alterations or modifications within proteins by analyzing regional changes in peptide coverage across the protein sequence. Rather than solely quantifying whole-protein abundance, PLF identifies localized differences in peptide yield, often indicative of proteolytic cleavage, confirmational change, or post-translational damage modifications.^28^ This technique has proven particularly valuable in matrix biology, where structural matrix proteins in diverse tissues are frequently subjected to selective enzymatic processing.^29–31^ In the context of kidney health, PLF was applied to investigate matrix remodeling in aging, and this revealed distinct regions within matrix proteins, including collagen I and collagen IV, that undergo structural modification.^32^ These region-specific patterns may also suggest targeted protease activity if correlated to known cleavage sites and support the hypothesis that fragmentation of matrix components may contribute to a circulating biomarker signature in kidney fibrosis.

Kidney fibrosis represents a pathological imbalance between matrix formation and degradation, leading to disordered matrix remodeling and ultimately, organ failure. Understanding mechanisms of kidney matrix protein turnover, how this is disrupted in the context of disease, and identifying matrix fragments produced could offer a non-invasive strategy for early detection and prevention of kidney fibrosis progression. To this end, we performed a comprehensive multimodal analysis of kidney matrix in Alport syndrome. Using ^13^C-lysine labeling, we generated the first proteome-wide turnover data for the kidney and discovered a molecular signature of altered abundance and turnover rates of individual kidney matrix proteins in Alport mice, suggesting uncoupling between matrix synthesis and degradation. With bioinformatic modeling, we predicted fragmentation of matrix proteins and, using targeted biomarker assays, detected altered levels of matrix fragments in individuals with Alport variants compared to healthy controls. Together, these findings demonstrate abnormal matrix dynamics in kidney disease, leading to the detection of circulating matrix associated with kidney matrix remodeling.

## Results

### Altered transcription of extracellular matrix genes in kidney disease

**Figure 1:**
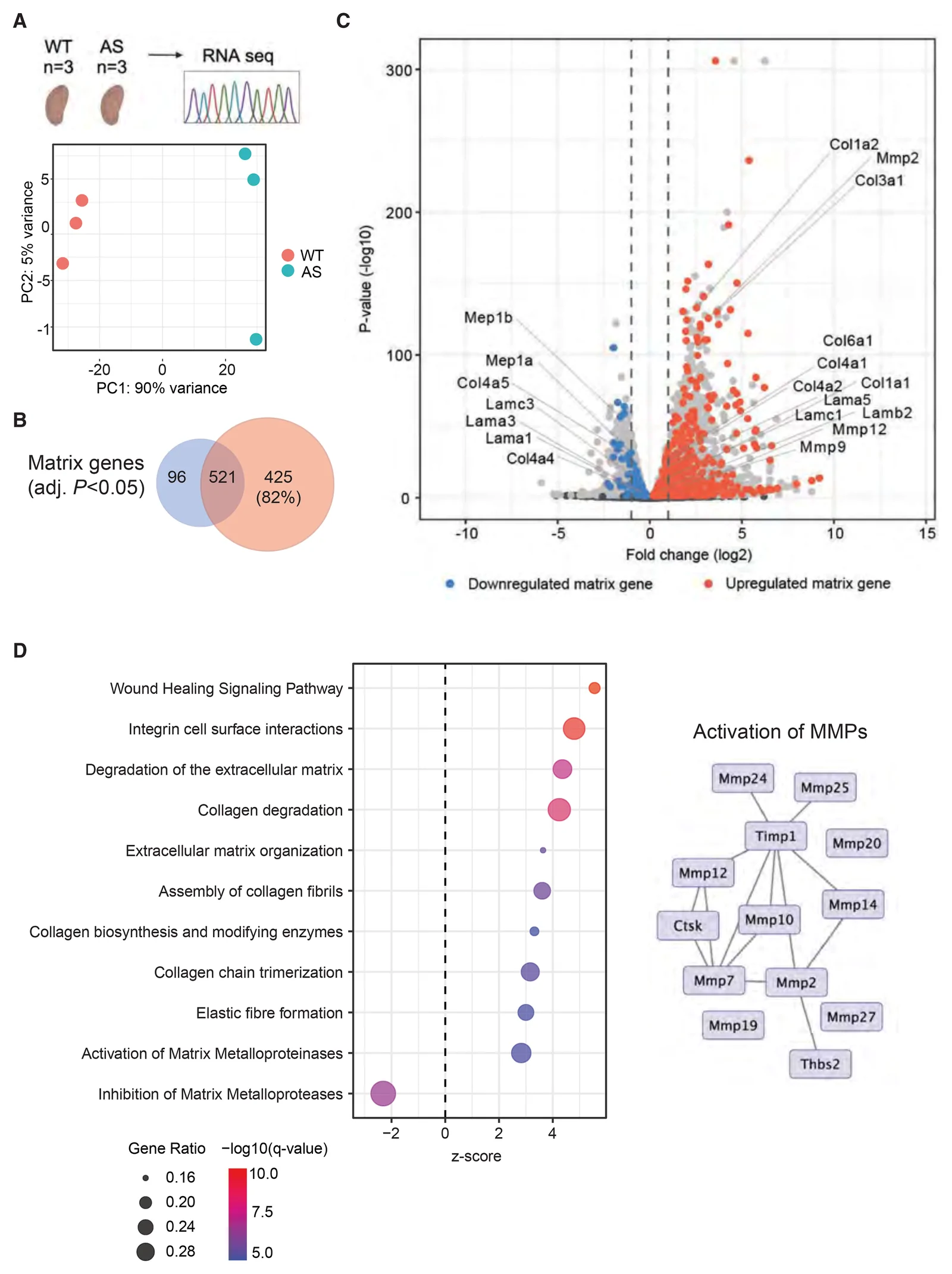
Upregulation of extracellular matrix genes and pathways in kidney disease (A)Experimental design for transcriptomic analysis of wild-type (WT; *n* = 3) and Alport (AS; *n* = 3) kidneys (upper) and principal-component analysis (PCA) of the transcriptome (lower). (B)Venn diagram of differentially expressed kidney matrix genes (adjusted *p* value < 0.05) in AS kidney. (C)Volcano plot of differentially expressed kidney matrix genes in AS versus WT kidney. Upregulated genes are shown in red, downregulated genes in blue, and non-matrix genes in gray. Dotted black line represents fold change greater or lesser than 2 at adjusted *p* value < 0.05. (D)Ingenuity pathway analysis of significantly upregulated kidney matrix genes showing clusters enriched for canonical pathways of interest (left) and network of significantly upregulated matrix regulators associated with activation of matrix metalloproteases (MMPs) (right). Nodes represent proteins, and connections represent interactions identified in STRING software.^34^

To investigate the genes and pathways affected in kidney disease progression, we used *Col4a5*^−/*Y*^ (Alport) mice, which harbor a genetic deletion of the critical exon 36, resulting in the absence of the collagen-α5(IV) protein.^16^ These mice phenocopy human Alport syndrome with abnormal glomerular basement membrane thickness, podocyte effacement, and progressive albuminuria.^16^ We confirmed significant albuminuria evident from 12 weeks, which was accompanied by progressive collagen accumulation and interstitial kidney fibrosis (Figures S1 and S2). We harvested kidneys from 16-week-old Alport and wild-type mice (*n* = 3 per group) and performed bulk RNA sequencing (Figure 1A). Principal-component analysis of the transcriptomic data demonstrated sample clustering by genotype (Figure 1A). Overall, we found 9,719 differentially expressed genes (adjusted *p* value<0.05) in the Alport kidneys. We further identified 521 extracellular matrix genes, as defined by MatrisomeDB,^33^ and the majority of these (82%) were upregulated in Alport kidneys (Figure 1B). With differential expression analysis, we found fibrillar collagens (collagen I and collagen III), collagen IV α1,α1,α2, and key components of the glomerular basement membrane, including constituent chains of the mature laminin 521 network, were upregulated (Figure 1C). Furthermore, a total of 94 extracellular matrix regulators important in kidney matrix remodeling, basement membrane component degradation, and collagen cross-linking were upregulated, including matrix metalloproteinases, ADAM and ADAMTS proteins, serine and cysteine proteases, and lysyl oxidases (Figure S3A). Collagen IV chains comprising the α3,α4,α5 network, which is essential for glomerular barrier function and is disrupted in Alport syndrome, were downregulated (Figure 1C). Membrane-bound metalloproteinases (meprin1a and meprin1b) important in proteolytic cleavage, turnover, and remodeling of extracellular matrix were down-regulated, suggesting imbalanced protease activity in Alport kidneys. To determine the biological pathways enriched by upregulated genes in Alport mice, we performed Reactome pathway analysis and identified multiple overrepresented pathways with an adjusted *p* value of <0.05 (Figure S3B). The most significantly enriched pathways relate to dysregulation of inflammatory and immune response within the kidney, including the complement cascade, Toll-like receptor activity, neutrophil degradation, and cytokine signaling. In addition, pathways linked to degradation of extracellular matrix and collagen turnover were dominant and enriched. In parallel, for a more comprehensive analysis and to gain mechanistic understanding, we conducted Ingenuity Pathway Analysis of the Alport transcriptome, and we found largely overlapping enrichment for matrix degradation along with upregulation of matrix metalloproteinase (MMP) activation and concurrent downregulation of MMP inhibition (Figure 1D). Taken together, we discovered altered transcription of immune system, extracellular matrix, and matrix degrading proteases, suggesting that abnormal matrix turnover is a feature of Alport kidney disease.

### Heavy isotope ^13^C-lysine pulse-labeling of the mouse kidney proteome

**Figure 2:**
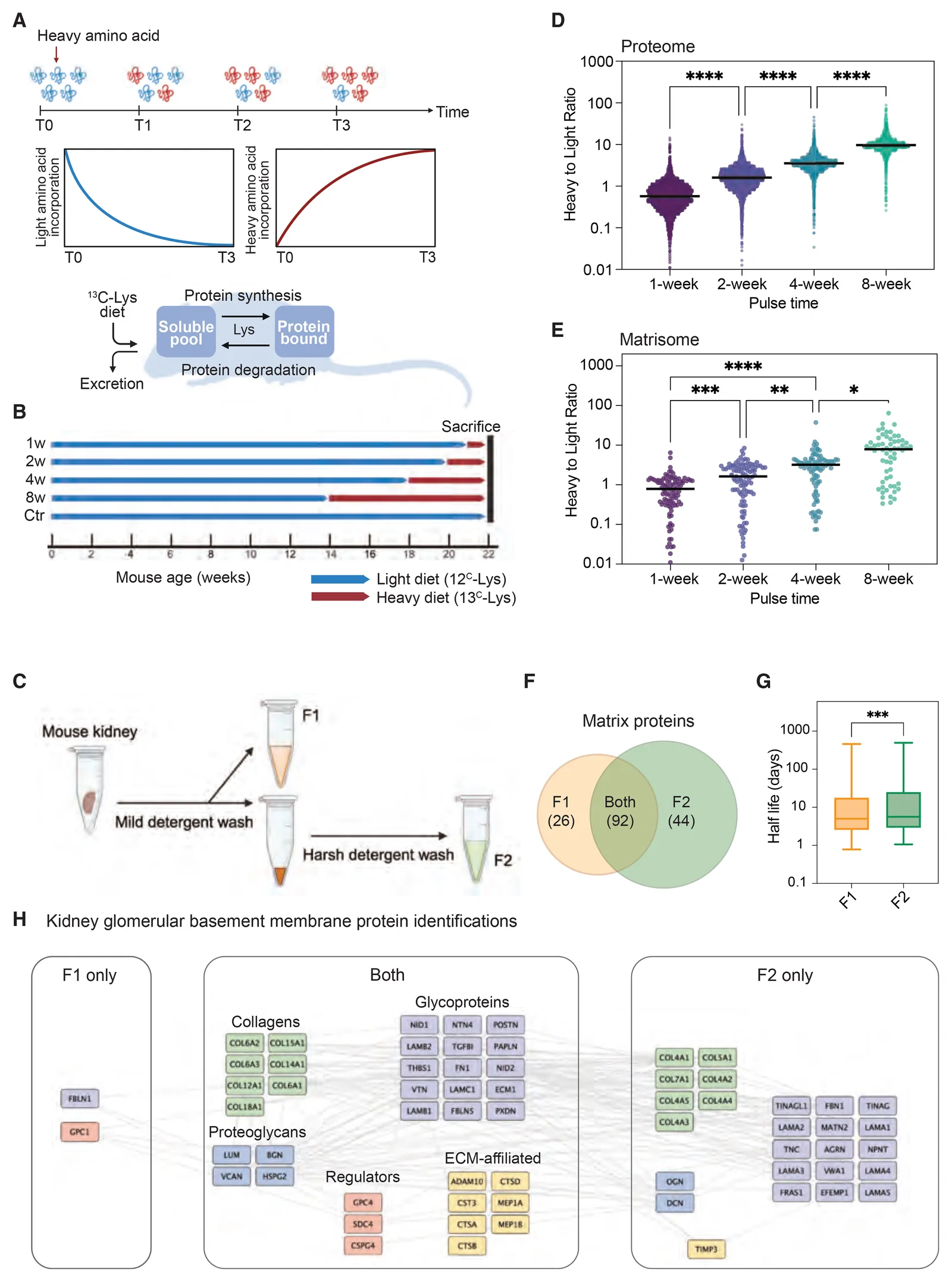
Heavy isotope pulse-labeling of the mouse proteome and identification of a ^13^C-lysine-labeled kidney matrisome (A)Schematic demonstrating the principle of ^13^C-lysine (^13^C-Lys) labeling in mammals (upper) and lysine pools that form the basis of modeling protein half-life estimates (lower).^35^ (B)Experimental design for ^13^C-Lys pulse-labeling used for half-life estimation. *N* = 3 biological replicates per condition. (C)Schematic of the two-step fractionation protocol for matrix-enrichment of mouse kidney. (D and E) Heavy-to-light ratios for all proteins (D) and matrix proteins (E) in kidney from mice fed ^13^C-Lys diet for 1, 2, 4, or 8 weeks. Bars show median. (F)Venn diagram of matrix protein identifications in each fraction. (G)Comparison of matrisome half-lives between fraction 1 (F1) and fraction 2 (F2). Bars show median with 95% confidence intervals. (H)Network of glomerular matrix protein identifications in F2, filtered using *basement membrane* BASE.^15^ Nodes are colored by matrisome group as defined by MatrisomeDB,^33^ and connections represent interactions identified by STRING software.^34^ Data are presented as median. ****p < 0.0001, ***p = 0.0006, **p = 0.0037, and *p = 0.0169 by Kruskal-Wallis test with correction for multiple comparisons (D and E). ***p = 0.0006 by Mann-Whitney test (G).

To investigate matrix protein turnover, we used heavy isotope labeling of mice. With this approach, the rate at which ^13^C-lysine-labeled amino acids from the diet are incorporated into newly synthesized proteins is used to calculate protein turnover rates (Figure 2A, upper panel). Mass spectrometry (MS)-based identification of peptides derived from biologically identical “heavy” and “light” versions of the same peptides allows quantification of pre-existing and newly synthesized proteins. Ratios between the MS signal from matched heavy and light peptides (heavy-to-light ratios) are then converted into estimates of protein half-life,^35^ which includes a “soluble pool” of free lysine within labeled animals to account for reuse of amino acids from protein degradation (Figure 2A, lower panel). To measure protein turnover in healthy adult kidneys, 22-week wild-type (C57BL/6) mice were pulse-labeled with a ^13^C-lysine heavy diet for 1, 2, 4, or 8 weeks (Figure 2B). The purpose of using multiple labeling intervals was to generate sufficient data for modeling of protein half-lives.^35^ Following pulse-labeling, kidneys were harvested, fractionated, and analyzed by MS. To facilitate enhanced matrisome coverage, we designed a two-step fractionation protocol (see STAR Methods) to generate a more soluble, cellular protein fraction (F1) and a less soluble, matrix-enriched fraction (F2) (Figure 2C). Throughout the labeling period, heavy-to-light ratios significantly increased across pulse-times throughout the proteome (Figure 2D) and matrisome (Figure 2E), confirming heavy isotope incorporation over time. The heavy label was incorporated into the matrisome at a lower rate, consistent with more longer-lived extracellular proteins, relative to intracellular proteins. Principal-component analysis of both F1 and F2 demonstrated sample clustering with pulse-time (Figure S4A). Overall, we identified 162 matrix proteins and, as expected, F2 was enriched by less soluble core matrisome proteins (Figure 2F). Matrix proteins identified in the more soluble F1 demonstrated significantly shorter half-lives than those identified in the less soluble F2 (Figure 2G). Given Alport syndrome represents an inherent defect of the GBM, we focused our analysis by searching protein identifications against *basement membrane*BASE, a comprehensive resource of basement membrane proteins.^15^ This revealed a network of matrix proteins identified across both fractions with enrichment of network-forming type IV collagens (COL4A1, COL4A2, COL4A3, COL4A4, and COL4A5) in F2 (Figure 2H). Conversely, fibril-associated collagens (COL14A1 and COL15A1), beaded filament collagens (COL6A1, COL6A2, and COL6A3), and the basement membrane component collagen XVIII (COL18A1) were detected in both fractions, reflecting the more soluble nature of loosely bound collagens. Overall, we identified the first ^13^C-lysine-labeled kidney proteome and established a pipeline to enable investigation of matrix turnover in kidney disease.

### Altered matrix composition in Alport kidney disease

**Figure 3:**
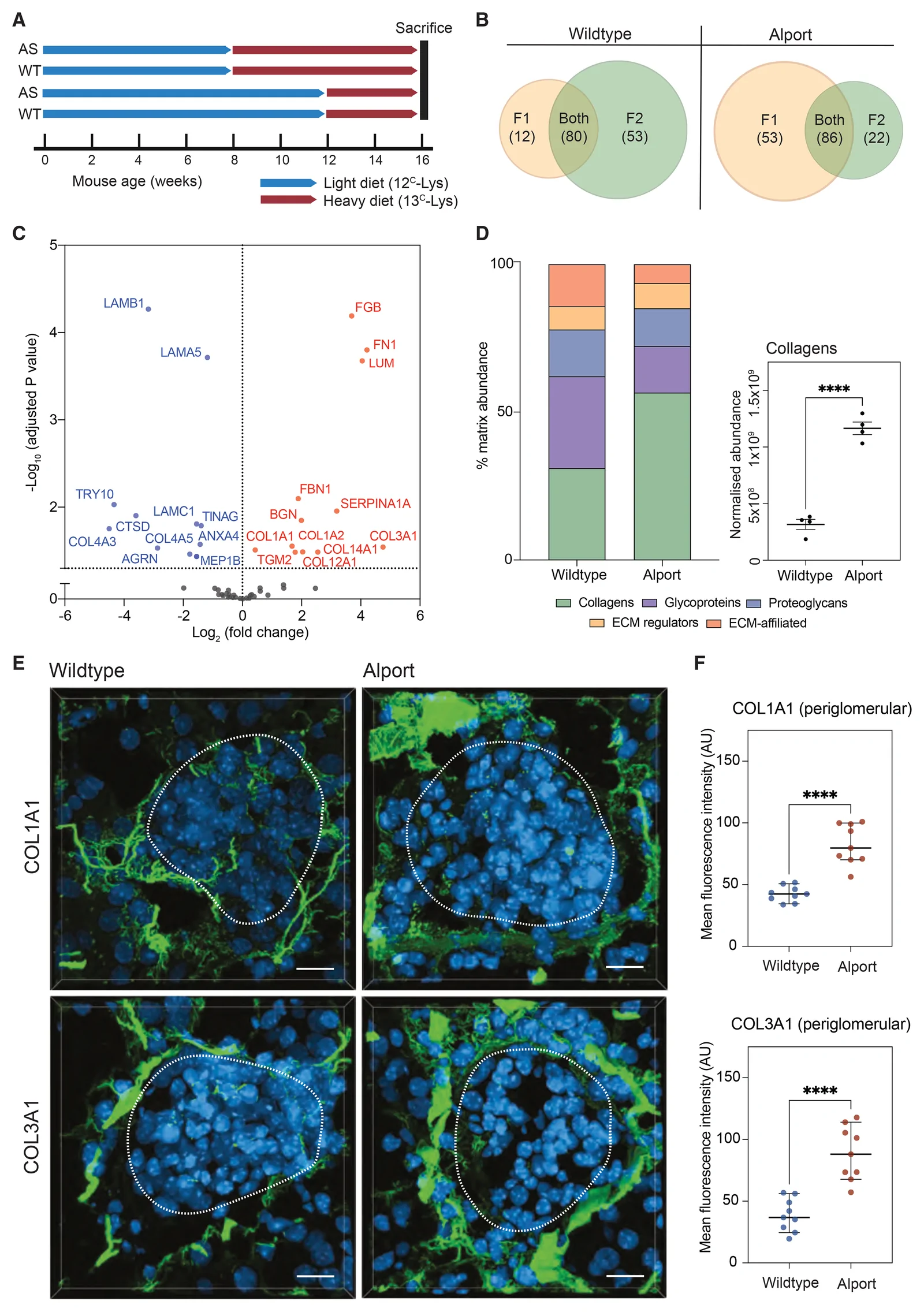
Matrix composition is altered in Alport mice with increased abundance of fibrillar collagens (A)Experimental design for ^13^C-Lys labeling of wild-type (WT) and Alport (AS) mice. *N* = 4 biological replicates per condition. (B)Venn diagrams of matrix protein identifications in fraction 1 (F1) and fraction 2 (F2) from WT and AS kidneys. (C)Volcano plot of fold change in abundance of kidney matrix proteins in F2 from AS versus WT kidney. Proteins with increased abundance in AS kidney are shown in red and those with decreased abundance in blue. Dotted black line represents significance threshold (adjusted *p* value < 0.05). (D)Distribution of matrisome protein abundance in F2 from WT and AS kidneys (left) and normalized abundance (to total matrix) of collagen (right). Bars show mean ± SEM. (E)Representative immunofluorescence images of COL1A1 (upper) and COL3A1 (lower) in WT and AS kidneys, signal shown in green. Glomeruli are encircled by white dotted line. Scale bars, 15 μm. (F)Quantification of periglomerular fluorescence intensity of COL1A1 (upper) and COL3A1 (lower). Bars show median with 95% confidence intervals. *N* = 3 biological replicates and *n* = 3 technical replicates. ****p < 0.0001 by parametric unpaired two-tailed *t* test (D) and ****p < 0.0001 by Mann-Whitney test (F).

Next, we investigated protein abundance and turnover in *Col4a5*^-/*Y*^ (Alport) mice. We pulse-labeled 16-week-old hemizygous male *Col4a5*^-/*Y*^ mice and wild-type littermate controls by feeding them a ^13^C-Lysine heavy diet for 4 or 8 weeks (Figure 3A). The purpose of using two pulse-labeling intervals was to generate additional pulse-time data for accurate modeling of half-lives using the model described above.^35^ Following pulse-labelling, whole kidney tissue was harvested, fractionated using the two-step extraction protocol described earlier, and analyzed by mass spectrometry. Principal-component analysis of normalized abundances from Alport and wild-type kidney demonstrated samples clustering by condition and by fraction (Figure S5A). The more soluble F1 was predominated by cellular proteins and demonstrated 650 differentially expressed proteins, of which 77% were increased in abundance in Alport mice (Figure S5B). Reactome pathway enrichment analysis of significantly upregulated proteins highlighted pathways implicated in the progression of kidney fibrosis (Figure S5C). The KEAP1-NFE2L2 pathway, which mediates NRF2 activation, serves as a protective antioxidant pathway in the kidney, and studies in animal models have demonstrated this pathway as a promising therapy for Alport syndrome.^36^ To explore the kidney matrix, we next focused on the less soluble F2 fractions generated from mice fed with 8-week pulses of the heavy ^13^C-lysine diet. We detected 145 and 161 matrix proteins in wild-type and Alport kidneys, respectively (Figure 3B). While wild-type kidneys demonstrated matrix enrichment in F2 following fractionation, there was a shift in Alport kidney, with more matrix proteins identified in F1, suggesting that the Alport kidney matrix is more soluble (Figure 3B). Indeed, chains of collagen I and collagen IV were identified in both fractions in Alport kidneys as opposed to solely in F1 in wild-type kidneys. Next, we compared abundance (combined heavy and light protein intensity) of individual matrix proteins in wild-type and Alport kidneys. We found significant changes in matrix protein abundance between wild-type and Alport mice (Figure 3C). Fibrillar collagens chains (COL1A1, COL1A2, and COL3A1) were upregulated in Alport kidneys, while the laminin 521 network components were downregulated. Furthermore, we observed an overall increase in relative abundance of core matrix proteins in Alport kidneys (86%) compared to wild type (77%). The core matrisome of healthy kidneys was evenly composed of collagens (31%) and glycoproteins (31%) (Figure 3D). In Alport kidneys, the matrix composition shifted toward a predominance of collagens, with collagen abundance increasing to 57%, which was higher than that observed in wild-type mice (*p* < 0.0001) (Figure 3D), while glycoprotein abundance decreased to 16%. Next, we performed immunofluorescence imaging of kidney sections harvested from wild-type and Alport mice to validate our proteomic data and to investigate spatial distribution of kidney collagens (Figure 3E). We found increased signals for collagen I (COL1A1) and collagen III (COL3A1) surrounding the glomeruli, suggesting periglomerular fibrosis (Figures 3F and S6) and supporting our findings from the proteomic study.

**Figure 4:**
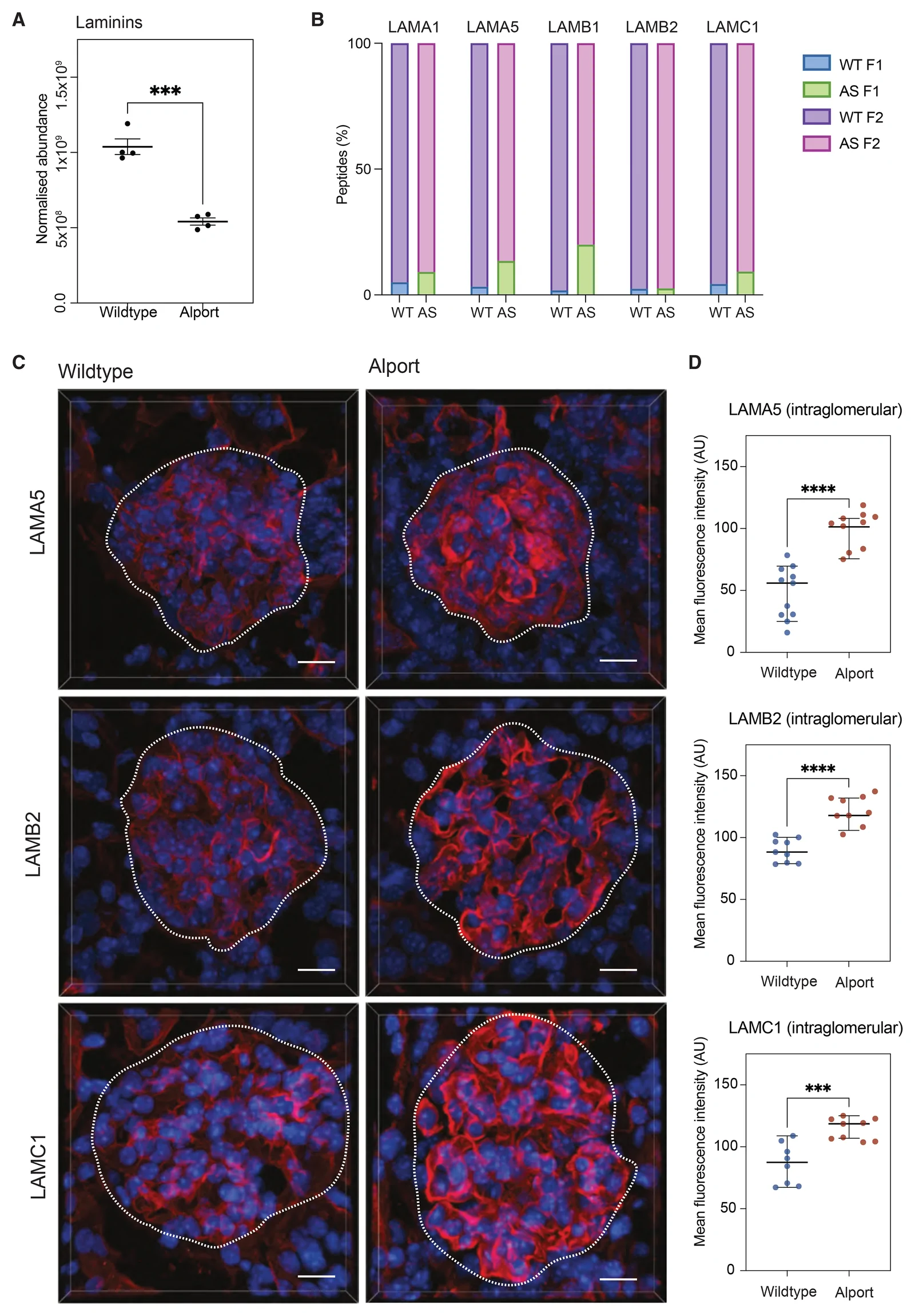
Altered solubility and distribution of laminin in kidney disease (A)Normalized abundance (to total matrix) of laminin in F2 from wild-type (WT) and Alport (AS) kidneys. Bars show mean ± SEM. (B)Laminin chain peptide counts identified in fraction 1 (F1) and fraction 2 (F2) from WT and AS kidney expressed as a percentage of total peptide count. (C)Representative immunofluorescence images of LAMA5 (upper), LAMB2 (middle), and LAMC1 (lower) in WT and Alport kidney sections; signal shown in red. Glomeruli are encircled by white dotted line. Scale bars, 15 μm. (D)Quantification of intraglomerular fluorescence intensity of LAMA5 (upper), LAMB2 (middle), and LAMC1 (lower). Bars show median with 95% confidence intervals. *N* = 3 biological replicates and *n* = 3 technical replicates. ***p = 0.0001 by parametric unpaired two-tailed *t* test (A). ****p < 0.0001 and ***p = 0.0010 by Mann-Whitney test (D).

For laminins, we observed decreased abundance in the less soluble F2 fraction from Alport compared to wild-type kidneys (*p* = 0.0001) (Figure 4A). To further explore the solubility of laminin chains, we quantified laminin peptides identified in F1 and F2 and observed relatively increased laminin peptides in F1 from Alport compared to wild-type kidneys (Figure 4B). Notably, this included the LAMA5 and LAMC1 chains of the glomerular laminin 521 network. Immunofluorescence imaging of kidney sections harvested from wild-type and Alport mice demonstrated increased intraglomerular signal for LAMA5, LAMB2, and LAMC1 (Figures 4C and 4D). Interestingly, the spatial distribution of laminin was altered, appearing disorganized and spreading outside the basement membrane, and this pattern correlated with the degree of solubility. That is, LAMA5, which demonstrated increased solubility in Alport kidney, lost spatial organization compared to LAMB2, which did not demonstrate increased solubility and retained typical organization. Collectively, the proteomic and imaging data indicate that matrix proteins are disrupted in Alport kidneys with increased solubility and evidence of structural disorganization.

### Rapid turnover of kidney matrix in disease

**Figure 5:**
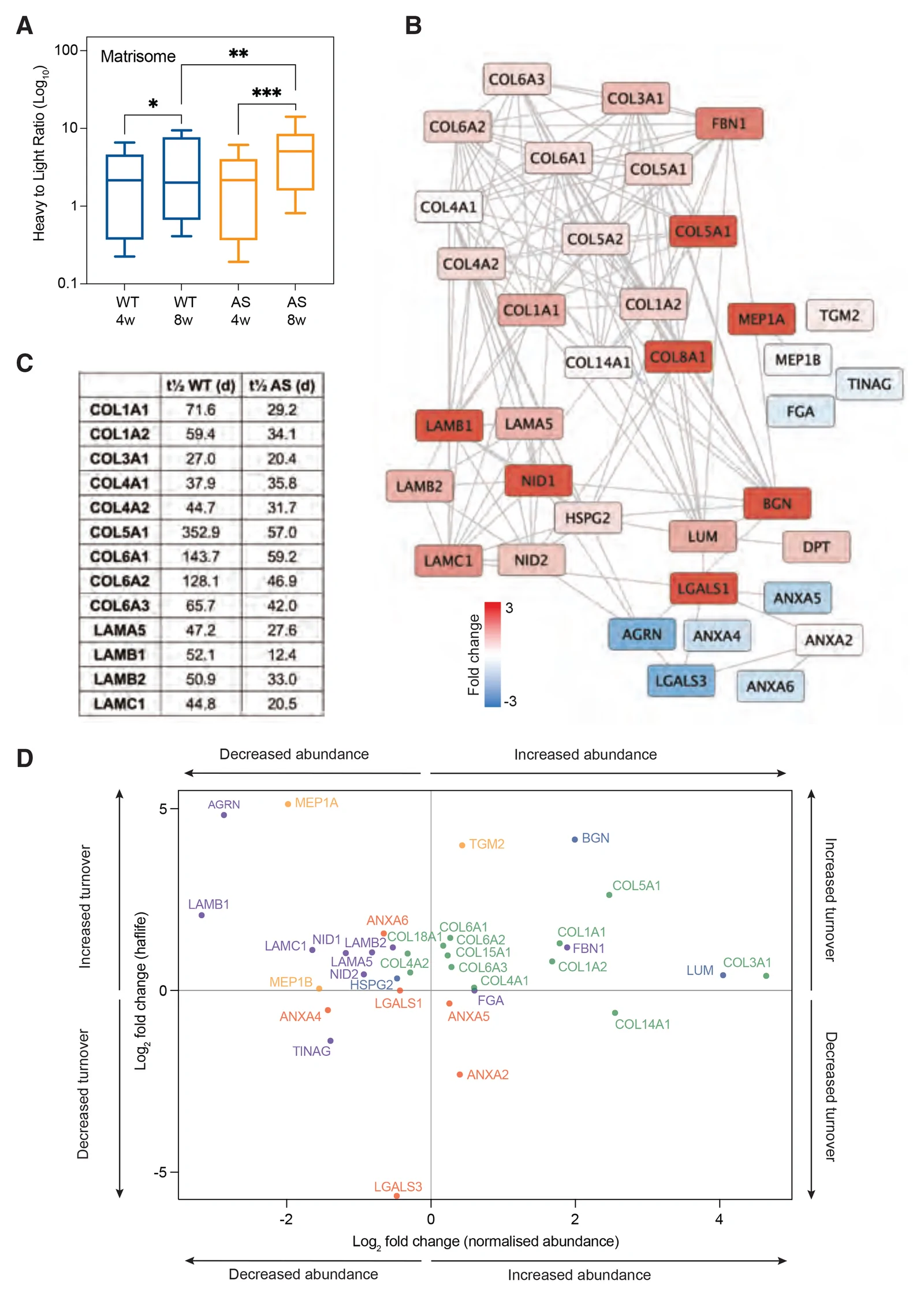
Matrix turnover is increased in kidney disease (A)Heavy-to-light ratios of kidney matrix proteins in F2 after 4- and 8-week ^13^C-Lys pulses in wild-type (WT) and Alport (AS) mice. Line at median and whiskers represent 10^th^–90^th^ centiles. (B)Protein-protein interaction network of matrix proteins with altered rates of ^13^C-Lys incorporation in AS kidney compared to WT. Nodes are colored by fold change in heavy-to-light ratio, and connections represent interactions identified by STRING software.^34^ (C)Half-life (t½) estimations (days) of key kidney collagens and laminins in WT and AS kidney determined by published mass spectrometry workflow.^35^ (D)Fold change in abundance and half-life of individual matrix proteins in AS compared to WT kidney. Proteins are colored by matrix category as per MatrisomeDB.^33^ *N* = 4 biological replicates per condition for all analysis. *p = 0.023, **p = 0.02, and ***p < 0.001 by two-way ANOVA of log_10_ transformed data (A).

The ^13^C-lysine labeling approach allowed us to study protein turnover, and we calculated heavy-to-light ratios and half-lives, for hundreds of kidney proteins (Table S1). Principal-component analysis of heavy-to-light ratios from F1 and F2 demonstrated segregation by condition (Figure S7A). Longer pulses of heavy diet led to significantly higher levels of ^13^C-lysine incorporation into the proteome, represented by higher heavy-to-light ratios after the 8-week pulse compared to 4-week pulse in both wild-type and Alport mice (Figure S7B), and this pattern was preserved for the matrisome alone (Figure 5A). For the matrisome proteins, there was significantly higher heavy-to-light ratios in Alport kidneys compared to wild type after 8 weeks of pulse labeling (1.7-fold increase, *p* = 0.002) (Figure 5A), suggesting that the effect of genotype is different at each time point (interaction *p* = 0.0169). As such, we used the 8-week pulse for further downstream analysis of kidney matrix turnover. To visualize changes in ^13^C-lysine incorporation of individual matrix proteins, we performed protein-protein network mapping and colored nodes according to their heavy-to-light ratio fold change in Alport kidneys compared to wild type (Figure 5B). Remarkably, we observed increased heavy-to-light ratios, indicating faster turnover, of all kidney collagens and key components of the glomerular basement membrane, including the laminin 521 chains, nidogens, and heparin sulfate proteoglycans. Indeed, half-lives of kidney collagens and laminins were universally shorter in Alport mice, compared to wild type (Figure 5C), indicating that the kidney matrix is rapidly turned over in Alport syndrome. Protein homeostasis depends on the fine balance between protein synthesis and degradation. In the steady state, these processes are balanced but disruption in steady-state protein dynamics causes abnormal tissue remodeling. Our data demonstrate a molecular signature of altered turnover rates of key kidney matrix proteins in Alport mice, and this pattern is accompanied by changes in protein abundance (Figure 5D). To examine regulatory mechanisms of protein expression and turnover, we integrated our transcriptomic and proteomic data (Figure S7C) and found significant changes in gene expression showed weak correlation with significant changes in protein abundance; however, divergent expression was noted for laminin chains, suggesting post-translational regulatory mechanisms. Proteins showing significant changes in both gene expression and protein turnover included kidney collagens, laminins, and nidogen, indicating that transcriptional upregulation is accompanied by accelerated protein degradation. Overall, we demonstrate several patterns of dysregulated kidney matrix dynamics, suggesting uncoupling between matrix synthesis and degradation, resulting in the excessive accumulation of disordered matrix in kidney disease.

### Detection of circulating matrix fragments in children with Alport syndrome

**Figure 6:**
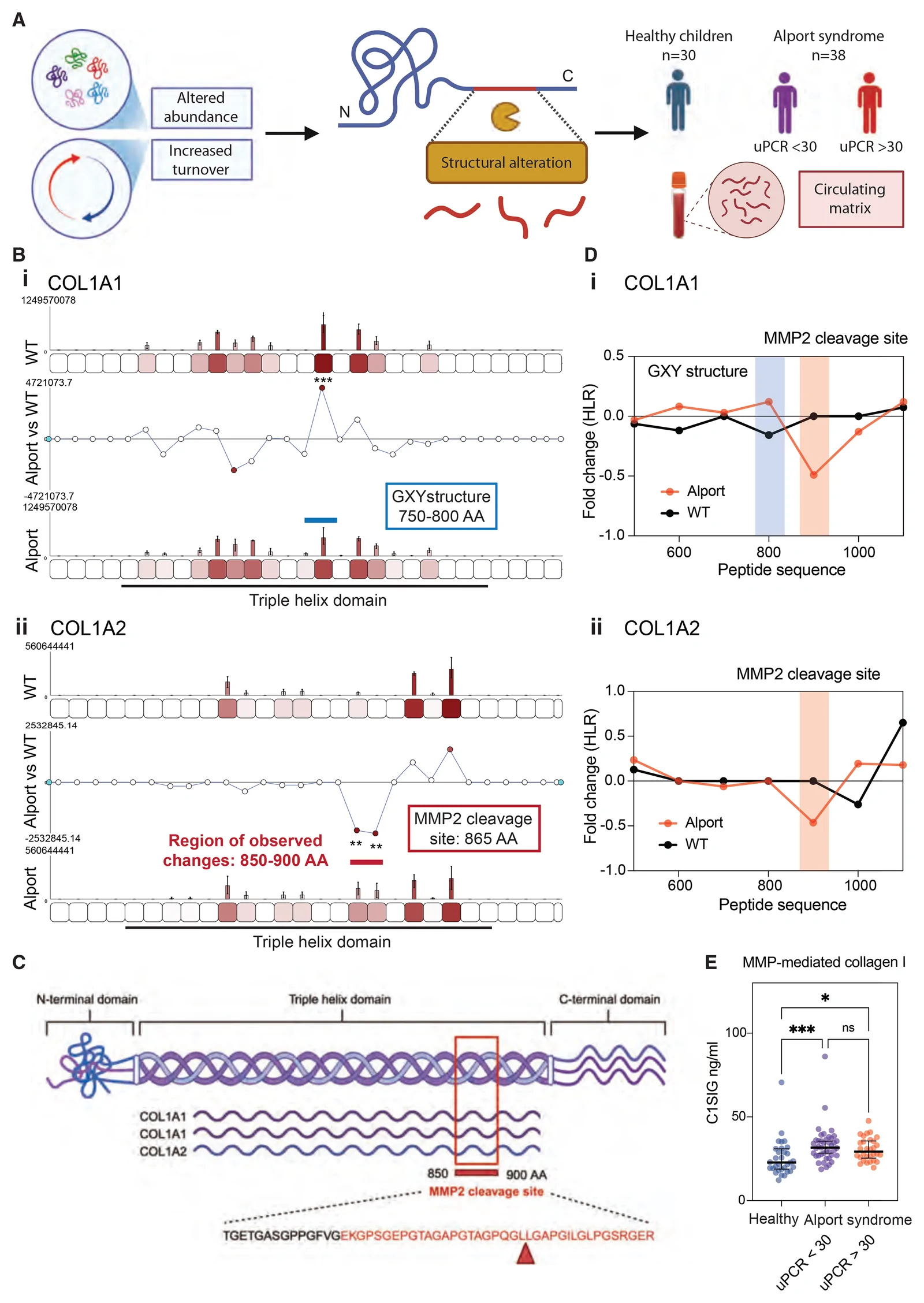
Structure-specific alterations in collagens in kidney disease (A)Schematic of the approach to identifying potential mechanisms of matrix fragment release and detection of circulating matrix. uPCR, urine protein creatinine ratio thresholds of <30 mg/mmol or >30 mg/mmol were used as proteinuria thresholds. (B)PLF analysis of COL1A1 and COL1A2 in wild-type and Alport kidneys (for PLF methodology see methods). Bar graphs = averaged, normalized peptide spectrum matches (PSMs) per segment; error bars = standard deviation. Line graph *y* axes = wild-type-Alport PSMs/segment length. (i) COL1A1 peptide yield differences coincided with the GXY structure of the triple helix domain (shown by blue bar). (ii) COL1A2 peptide yield differences coincided with a known MMP2 cleavage site (shown by red bar). *N* = 4 biological replicates per condition. (C)Schematic of collagen(I)-α1-α1-α2 trimer demonstrating structural domains and the MMP2 cleavage site identified in Alport kidney. (D)Fold change in heavy-to-light ratio (HLR), normalized to average turnover for the total protein, at 100AA segments, which demonstrated peptide yield differences by PLF analysis in (i) the GXY structure of the triple helix domain (affected segment highlighted in blue) and (ii) the MMP2 cleavage site (affected segment highlighted in red). *N* = 4 biological replicates per condition. (E)Circulating MMP-mediated collagen I fragments in serum from healthy children (blue dots, *n* = 30) and children with Alport syndrome (*n* = 38) with (red dots) and without proteinuria (purple dots). Bars show median with 95% confidence intervals. *p < 0.05, **p < 0.01, and ***p < 0.001 by unpaired Bonferroni-corrected, repeated measures ANOVA (B). ***p = 0.0003 and *p = 0.0111 by Kruskal-Wallis test with correction for multiple comparisons (E).

Having identified matrix proteins with increased turnover and altered abundance in Alport kidneys, we next applied peptide location fingerprinting (PLF) to predict matrix proteins with structure-associated differences/modifications and correlated these to known MMP cleavage sites to gain mechanistic insight into potential proteolytic cleavage and fragment release (Figure 6A). We then screened for predicted matrix fragments in serum from healthy children, and children with Alport variants, with and without proteinuria. Using PLF, we identified 40 matrix proteins with significant structure-associated differences between wild-type and Alport kidneys (Figure S8A). Of these, over 50% were associated with basement membranes, indicating substantial structure-associated remodeling in Alport compared to wild-type kidneys. Given our earlier findings with collagen I, it was notable that PLF showed peptide yield differences in COL1A1 peptides at amino acids 750–800 corresponding to the G-X-Y amino acid repeat structure, which is key for triple helix formation and flexibility^37^ (Figure 6B). In COL1A2, peptide yield differences were identified at amino acids 850–900 corresponding to a known MMP-2 cleavage site at position 865^38^ (Figure 6B). This predication is based on identification of the intact fragment containing the MMP-2 cleavage site in Alport but not wild-type kidneys, suggesting altered susceptibility to MMP2 cleavage in Alport kidneys (Figure 6C). This hypothesis is further supported by differences in turnover of peptides along COL1A1 and COL1A2 sequences. Both chains contained specific protein regions demonstrating slower turnover of peptides corresponding to the COL1A2 MMP2 cleavage site among amino acids 850–950, supporting potential altered availability for proteolytic cleavage in Alport kidney (Figures 6D; Table S2). As COL1A1 and COL1A2 trimerize to form the collagen I α1,α1,α2 heterotrimer, it is likely that altered susceptibility to MMP2 cleavage affects both constituent chains (Figure 6C). To investigate the extent of post-translational modifications in Alport kidney in greater detail, we focused on collagen type I and widened our MS search parameters to capture semi-tryptic peptides. Alport mice showed significantly more semi-tryptic COL1A1- and COL1A2-derived peptides compared to wild-type mice (Figure S9A), indicative of increased levels of endogenous degradation or fragmentation. To directly visualize increased proteolytic activity in Alport kidney, we used DQ collagen I substrate, which generates fluorescent fragments upon *in situ* collagenase activity, and we found significantly higher levels within Alport glomeruli (Figure S9B). Next, using targeted biomarker assays (Nordic Biosciences, Denmark), we searched for collagen 1 fragments in serum from healthy children and children with Alport variants (for study participant characteristics, see Figure S5B). The C1SIG biomarker assay quantifies neo-epitope fragments of human collagen I-α1 that are generated through MMP-mediated cleavage of the C-terminal end of the helix, serving as a marker of matrix remodeling. In keeping with predictions from our data, MMP-mediated collagen 1 fragments were increased in Alport syndrome, compared to healthy controls (*p* = 0.0003) (Figure 6E). Remarkably, circulating collagen I fragments were detected prior to the onset of significant proteinuria (urine protein creatinine ratio <30mg/mmol) indicating that cleaved matrix can be detected in the circulation before the onset of overt kidney damage. To support this, we also detected elevated circulating levels of MMP-mediated collagen degradation fragments, using the C1M (collagen type I)^39^ and rCTX-III (collagen type III)^40^ assays, in blood samples from 16-week-old Alport mice, compared to wild type (Figure S9C).

**Figure 7:**
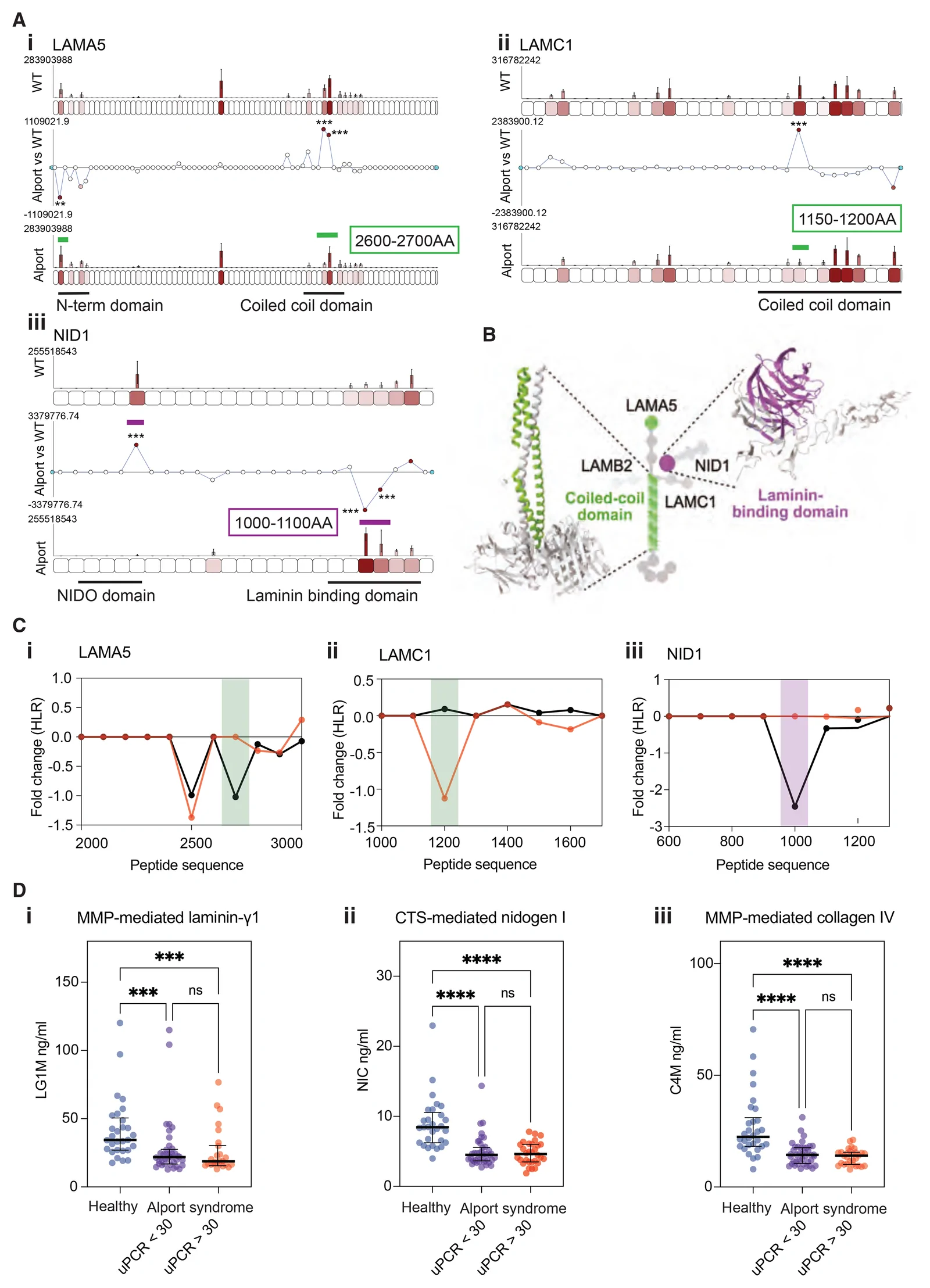
Basement membrane proteins are structurally altered in kidney disease (A)PLF analysis of LAMA5, LAMC1, and NID1 in wild-type and Alport kidneys (for PLF methodology see methods). Bar graphs = averaged, normalized peptide spectrum matches (PSMs) per segment; error bars = standard deviation. Line graph *y* axes = wild-type-Alport PSMs/segment length. (i) LAMA5 and (ii) LAMC1 peptide yield differences coincided with the coiled-coil domain. (iii) NID1 peptide yield differences coincided with the laminin-binding domain. *N* = 4 biological replicates per condition. (B)Schematic of LAMA5 and LAMC1 within the laminin 521 quaternary structure showing disease-affected coiled-coil regions (green) and the disease-affected laminin-binding domain of NID1 (purple). Protein crystal structures were downloaded from the Protein Data Bank (PDB5XAU). (C)Fold change in heavy-to-light ratio (HLR), normalized to average turnover for the total protein, at 100AA segments, which demonstrated peptide yield differences by PLF analysis in (i) LAMA5 coiled-coiled domain (affected segment highlighted in green), (ii) LAMC1 coiled-coiled domain (affected segment highlighted in green), and (iii) NID1 laminin-binding domain (affected segment highlighted in purple). *N* = 4 biological replicates per condition. (D)Circulating matrix fragments in serum from healthy children (blue dots, *n* = 30) and children with Alport syndrome (*n* = 38) with (red dots) and without proteinuria (purple dots), showing (i) MMP-mediated laminin-γ1 fragments, (ii) cathepsin (CTS)-mediated nidogen 1 fragments, and (iii) MMP-mediated collagen IV fragments. Bars show median with 95% confidence intervals. *p < 0.05, **p < 0.01, and ***p < 0.001 by unpaired Bonferroni-corrected, repeated measures ANOVA (A).****p < 0.0001 and ***p = 0.0006 by Kruskal-Wallis test with correction for multiple comparisons (D).

With proteomics, we also found that laminin chains had decreased overall abundance, with increased solubility, altered basement membrane localization, and increased turnover in Alport kidneys. Using PLF, we identified structure-associated differences in several laminin chains (Figure S8A), including LAMA5 and LAMC1. Both chains had differences in the C-terminal end of the coiled coil long arm of laminin 521 (Figure 7A). The quaternary arrangement and structure of the laminin coiled coil domain is important not only for structural stability of the laminin 521 heterotrimer but also for integrin binding^41^ (Figure 7B). Nidogen 1, a cross-linking glycoprotein localized close to the collagen IV network of the GBM,^9^ demonstrated structural differences within the laminin-binding domain (Figure 7A). Here, LAMC1 further stabilizes the basement membrane through interaction with nidogen, at the laminin-binding domain, to bridge the laminin 521 and collagen IV networks.^42–44^ The differences observed within these regions may therefore provide the molecular basis for disease-related disruption in laminin 521 anchorage within the basement membrane, increasing solubility and breakdown. Regions predicted by PLF to display structure-associated differences also demonstrated changes in turnover rates of corresponding protein segments, reinforcing that these functional domains are likely implicated in basement membrane remodeling in Alport kidneys (Figures 7C; Table S2). Next, we again searched for related matrix fragments in serum from healthy children and children with Alport variants using targeted biomarker assays (Nordic Bioscience, Denmark). The LG1M^45^ and C4M^46^ biomarker assays measure fragments released by MMP-mediated cleavage of laminin-γ1 and collagen IV, respectively, while the NIC^47^ biomarker assay measures fragments released by cathepsin-S-mediated cleavage of nidogen 1. Interestingly, we detected decreased levels of circulating laminin-γ1, nidogen 1, and type IV collagen fragments in children with Alport variants, before the onset of proteinuria (Figure 7D). Collectively these data demonstrate prediction and subsequent detection of circulating matrix fragments that both provide mechanistic insight into basement membrane remodeling and fibrosis and have potential as disease-specific biomarkers.

## Discussion

Accumulation of extracellular matrix is the hallmark of kidney fibrosis,^11,48^ yet the dynamic balance between matrix synthesis and degradation *in vivo* remains poorly understood. In this study, we conducted a multimodal investigation of kidney matrix homeostasis by integrating transcriptomic profiling, heavy isotope-labeled proteomics, peptide location fingerprinting, and targeted biomarker detection to understand the processes that drive abnormal matrix remodeling in kidney disease. We discovered disruption in matrix composition, solubility, and turnover and potential proteolytic cleavage and macromolecular interactions, reflecting pathological matrix remodeling in kidney disease. Furthermore, we found that predicted matrix fragments were detectable with altered abundance in patient and mouse serum samples raising prospects for disease-specific biomarkers of fibrosis.

Using bulk transcriptomic analysis to identify differentially expressed genes, we found upregulation of matrisome-encoding genes in Alport kidneys (82% of differentially expressed matrix genes) and enrichment of matrix assembly and degradation pathways. We observed downregulation of collagen IV α3,α4,α5 transcripts, which are reduced in Alport syndrome. Compensatory upregulation of collagen IV α1,α1,α2 transcripts and increased fibrillar collagens (Col1a1 and Col3a1) likely reflects a shift toward a fibrotic matrix phenotype.^49,50^ This is supported by increased expression of MMPs, ADAMTS proteases, and lysyl oxidases, which have established roles in matrix remodeling, collagen cross-linking, and degradation in fibrotic kidney disease.^51^ In keeping with previous findings in a *Col4a3* knockout Alport mouse, mRNA levels of membrane-bound MMPs meprin-α and meprin-β were significantly downregulated.^52^ Taken together, the transcriptomic data suggest dysregulated proteolysis, resulting in an imbalance between matrix synthesis and degradation in kidney disease.

To investigate the processes driving matrix remodeling at the protein level, we used heavy ^13^C-lysine pulse-labeling and deep proteomic profiling to quantify alterations in matrix abundance and turnover in *Col4a5*^-/*Y*^ (Alport) and wild-type mice. This dataset is the first heavy isotope-labeled kidney proteome, and it provides temporal resolution of matrix protein dynamics *in vivo*. The Alport kidney matrisome had significantly increased incorporation of heavy label, indicating increased turnover, which was especially evident for fibrillar collagens and components of the glomerular basement membrane, including the laminin 521 chains. Half-lives of key kidney collagens decreased from a median of 66 days in wild-type mice to just 36 days in Alport mice. Similarly, laminins had shorter half-lives, decreasing from a median of 55 days in wild-type to 26 days in Alport mice. Half-lives of matrix-associated proteins were shorter still but similar between wild-type and Alport mice, with a median of 5 days. The presence of such a range of turnover within the kidney matrix, yet with clear distinction among the matrix groups, suggests a tightly coordinated mechanism for maintaining matrix integrity and function. However, accelerated turnover of normally long-lived core matrix components in Alport kidneys implies either increased synthesis in response to damage or increased proteolytic activity leading to abnormal matrix remodeling.

Matrix composition and solubility changed dramatically in Alport kidneys. Collagens and laminins were re-distributed to the more soluble fraction, suggesting a loss of structural integrity and reduced incorporation into stable matrix networks. Supporting this, immunofluorescence imaging demonstrated disorganized laminin expression and periglomerular expansion of fibrillar collagens. These changes were accompanied by a shift in the overall composition of the Alport matrisome, with increased abundance of collagens. Accumulation of fibrillar collagen is a hallmark of organ fibrosis,^53^ including fibrosis associated with kidney disease^54^; however, the biological processes responsible for its excessive deposition are not understood. Interestingly, *de novo* production of collagen type I was observed in a *Col4a3* Alport mouse model^55^ and in a model of diabetic kidney disease,^56^ and inhibition of type I collagen accumulation reduced kidney fibrosis and improved kidney function. Our data support the inhibition of type I collagen synthesis as a putative therapeutic target to reduce Alport kidney fibrosis. In addition to increased collagens, we observed an overall reduction in basement membrane components, including laminins, in keeping with previous proteomic profiling.^16^ Importantly, the abnormal localization and increased solubility of laminin 521 chains, combined with their increased rates of turnover, indicate disrupted assembly or anchorage of this essential network. In Alport GBM, laminin subunit α2 deposition has been shown to be injurious to podocytes and promotes glomerular disease progression.^57–59^ Previous mass spectrometry analysis of glomeruli from *Col4a5*^-/*Y*^ mice detected low levels of glomerular laminin-α2,^16^ and in keeping with this, we detected laminin-α2 peptides in Alport samples but not in wild type; however, we were unable to quantify changes in peptide abundance or turnover due to incomplete coverage across samples. The importance of laminins in kidney disease was also demonstrated with an immunoassay targeting a neoepitope fragment of laminin-γ1, which found that laminin-γ1 remodeling was associated with a higher risk of adverse outcomes in people with kidney disease.^45^ Given the role of the laminin-nidogen-collagen IV network in maintaining integrity of the glomerular basement membrane,^1,60^ the structural instability of these components likely contributes to filtration barrier dysfunction and proteinuria observed in Alport syndrome.

To gain mechanistic insight into matrix remodeling in Alport mice, we applied peptide location fingerprinting, a bioinformatic prediction tool that detects regional structure-associated across protein sequences to predict processes such as proteolytic susceptibility. We integrated PLF-identified structural differences with cleavage site mapping and regional turnover data, to predict disease-associated matrix fragmentation. This approach not only advances our mechanistic understanding of matrix turnover but also informs the selection of candidate biomarkers for diagnostic assays.^29,32^ With PLF, we identified structural alterations in over 40 matrix proteins, more than half of which were known basement membrane components. Laminin chains, namely LAMA5 and LAMC1, had structural alteration at the C-terminal end of their coiled coil domains, which mediate heterotrimer stabilization and integrin binding.^41^ Nidogen-1 had structural alteration within the laminin-binding domain, suggesting that disruption of the laminin-nidogen-collagen IV interface^1^ contributed to basement membrane defects in Alport kidneys. As a readout of fibrosis markers, we found that collagen I chains (α1,α2) had increased peptide yield in regions corresponding to a known MMP-2 cleavage site,^38^ which was identified intact in Alport kidney, but not in wild type. Notably, these segments also had slower turnover, indicating structural changes in the quaternary structure of collagen 1 heterotrimers infers altered susceptibility to MMP-mediated proteolysis. These region-specific structural changes likely impair normal matrix assembly and allow for increased solubility and degradation. Proteolytic cleavage of collagen I in Alport kidney was supported by proteomic evidence of increased semi-tryptic collagen-I-derived peptides and enhanced *in situ* collagenase activity in kidney tissue sections.

To translate our findings into the clinical setting, we used targeted biomarker assays to detect circulating matrix fragments in the serum from children with Alport gene variants. We identified elevated levels of MMP-mediated collagen I fragments (C1SIG) in children with Alport variants prior to the onset of proteinuria, suggesting that matrix degradation precedes overt signs of glomerular injury. Circulating collagen degradation fragments were also raised in Alport mice compared to wild type. These data provide evidence that kidney matrix remodeling begins early in kidney disease and can be measured non-invasively. Conversely, we observed decreased levels of specific laminin-γ1 (LG1M), nidogen-1 (NIC), and collagen IV (C4M) fragments in Alport serum. This paradoxical reduction may reflect impaired cleavage due to disrupted matrix assembly or altered protease accessibility. Nonetheless, our data demonstrate that key laminin and collagen IV networks are degraded in disease, and this is detectable in the circulation before overt kidney injury.

Overall, we report patterns of abnormal matrix dynamics in kidney disease with increased solubility, accelerated turnover, structural instability, and the release of circulating matrix fragments. These fragments have the potential to serve as sensitive and specific biomarkers of kidney fibrosis in Alport syndrome. Further studies are required to determine the biological roles of matrix fragments in tissues both adjacent and distant from sites of basement membrane remodeling and fibrosis.

### Limitations of the study

Several limitations should be considered when interpreting the findings of this study. While transcriptomic and proteomic analyses were performed on bulk kidney tissue, which limits the resolution of glomerulus-specific changes when interpreting findings related to glomerular basement membrane proteins, immunofluorescence imaging confirms quantifiable differences in matrix protein signal within and surrounding glomeruli. As is standard for bottom-up proteomics using trypsin digestion, peptides containing arginine are not captured, and future studies employing complementary enzymes such as LysC may provide additional sequence coverage. Peptide location fingerprinting predictions require further experimental validation to confirm that specific cleavage events occur *in vivo*. Finally, the serum biomarker data were obtained from a relatively small pediatric cohort, and large-scale prospective validation studies are required to establish their clinical utility.

## Resource Availability

### Lead contact

Requests for further information and resources should be directed to and will be fulfilled by the lead contact, Rachel Lennon (rachel.lennon@ manchester.ac.uk).

### Materials availability

This study did not generate new unique reagents.

### Data and code availability

Transcriptomic data are available using the ArrayExpress accession number E-MTAB-15467. Proteomics data have been deposited to the ProteomeXchange Consortium via the PRIDE partner repository with the identifier PXD067091. Any additional information required to reanalyze the data reported in this paper is available from the lead contact upon request.

## Star Methods

### Key Resources Table

**Table:** 

REAGENT or RESOURCE	SOURCE	IDENTIFIER
Antibodies		
Rabbit polyclonal anti-collagen alpha-1(I) (Clone LF-68) 1:200 dilution	Kerafast	Cat#ENH018-FP
Rabbit multiclonal anti-collagen alpha-1(III) (Clone RM1028) 1:200 dilution	Abcam	Cat#ab283694
Rat monoclonal anti-laminin alpha-5 1:250 dilution	Sekiguchi Lab (Osaka University)	Cat#M5N8-C8, Manabe et al.^61^
Rat monoclonal anti-laminin beta-2 1:250 dilution	Sekiguchi Lab (Osaka University)	Cat#B24-N8-D6, Manabe et al.^61^
Rat monoclonal anti-laminin gamma-1 1:250 dilution	Sekiguchi Lab (Osaka University)	Cat#C11-N10-A10, Manabe et al.^61^
Rat monoclonal anti-perlecan (Clone A7L6) 1:500 dilution	Merck/Millipore	Cat#MAB1948P, RRID:AB_10615958
Donkey anti-Rabbit IgG Alexa Fluor 488 1:2000 dilution	Invitrogen	Cat#A21206, RRID:AB_2535792
Donkey anti-Rat IgG Alexa Fluor 594 1:2000 dilution	Invitrogen	Cat#A21209, RRID:AB_2535795
Biological samples		
Human serum (healthy controls)	Discovery Life Sciences	16/NW/0119
Human serum (Alport patient cohort)	Royal Manchester Children’s Hospital	N/A
Chemicals, peptides, and recombinant proteins		
OCT compound	PFM medical	Cat#PRC/OCT
Xylene	Fisher Chemical	Cat#X/0200/17
Ethanol 100%	Fisher Chemical	Cat#E/0650DF/P17
Direct Red 81	Sigma-Aldrich	Cat#195251-25G
Picric acid 2.1% aqueous solution	Atom Scientific	Cat#RRSP143-F
Acetic acid	Atom Scientific	Cat#RRSP171-E
Pertex mounting medium	Atom Scientific	Cat#RRSP008-11
TRIzol	Invitrogen	Cat#15596026
Chloroform	Fisher Chemical	Cat#C/4960/17
Isopropanol	Fisher Chemical	Cat#P-7500-24
Phosphate buffered saline (PBS)	Sigma-Aldrich	Cat#D8537
Sodium laurate	Sigma-Aldrich	Cat#L9755
Sodium deoxycholate	Sigma-Aldrich	Cat#D6750
Ammonium bicarbonate	Sigma-Aldrich	Cat#09830
Dithiothreitol (MS grade)	Sigma-Aldrich	Cat#D0632
Protease inhibitors	Sigma-Aldrich	Cat#11836170001
Phosphate inhibitors	Sigma-Aldrich	Cat#524629
Iodoacetamide (MS grade)	Sigma-Aldrich	Cat#I6125
Sodium chloride	Thermo Fisher Scientific	Cat#5/3160/65
Sodium dodecyl sulfate	Sigma-Aldrich	Cat#L4509
Triethylammonium bicarbonate	Sigma-Aldrich	Cat#T7408
Methanol (MS grade, Optima LC/MS)	Fisher Chemical	Cat#10767665
Phosphoric acid (H_3_PO_4_)	Acros Organics	Cat#463861000
Methyl *tert*-butyl ether (MTBE)	Chem Lab	Cat#Cl003991
Trypsin (MS grade)	Promega	Cat#V5111
Formic acid (Optima LC/MS)	Thermo Fisher Scientific	Cat#A117-50
Acetonitrile (Optima LC/MS)	Thermo Fisher Scientific	Cat#A955-1
Fish Skin Gelatin	Sigma-Aldrich	Cat#G7041-100G
Triton X-100	Sigma	Cat#X100-500ML
Tween 20	Sigma-Aldrich	Cat#P7949-500ML
Hoechst 33258 solution	Sigma-Aldrich	Cat#94403-1ML
ProLong Gold Antifade mountant	Invitrogen	Cat#P36934
DQ collagen type I fluorescein conjugate	Invitrogen	Cat#D-12060
Acetone	Fisher Chemical	Cat#A/0600/PC17
EDTA (Molecular Biology Grade)	Sigma-Aldrich	Cat#324506-100ML
Critical commercial assays		
Mouse Albumin ELISA Quantification Set	FORTIS Life Sciences	Cat#NC0096469
Creatinine Assay Kit	Bio-Techne R&D Systems	Cat#KGE005
RNeasy Mini Kit	QIAGEN	Cat# 74104
EnzChek Gelatinase/Collagenase Assay Kit	Invitrogen).	Cat#E12055
Pierce Rapid Gold BCA Protein Assay Kit	Thermo Fisher Scientific	Cat#A53225
Deposited data		
Transcriptomic data	ArrayExpress	E-MTAB-15467
Proteomics data	ProteomeXchange Consortium (PRIDE partner repository)	PXD067091
Experimental models: Organisms/strains		
C57BL/6J mice	Charles River	RRID:IMSR_JAX:000664
*Col4a5*^–/^ mice	International Mouse Phenotyping Consortium	Dickinson et al.^62^
Software and algorithms		
QuPath (V0.6.0)	https://qupath.github.io/	RRID:SCR_018257
Trimmomatic (v0.39)	https://www.usadellab.org/cms/index.php?page=trimmomatic	RRID:SCR_011848
STAR (v2.7.7a)	https://code.google.com/p/rna-star/	RRID:SCR_004463
DESeq2 (v1.40.2)	https://bioconductor.org/packages/release/bioc/html/DESeq2.html	RRID:SCR_015687
clusterProfiler (v4.8.3)	https://bioconductor.org/packages/release/bioc/html/clusterProfiler.html	RRID:SCR_016884
Enrichr (v3.2)	https://amp.pharm.mssm.edu/Enrichr/	RRID:SCR_001575
ReactomePA (v1.44.0)	https://bioconductor.org/packages/ReactomePA/	RRID:SCR_019316
Ingenuity Pathway Analysis (QIAGEN)	https://www.ingenuity.com/products/pathways_analysis.html	RRID:SCR_008653
Proteome Discoverer (v3.2)	https://www.thermofisher.com/order/catalog/product/IQLAAEGABSFAKJMAUH	RRID:SCR_014477
Perseus (v2.0.11.0)	https://www.perseus-framework.org	RRID:SCR_015753
MATLAB	https://www.mathworks.com/products/matlab/	RRID:SCR_001622
STRING (v11.5)	https://string.embl.de/	RRID:SCR_005223
Cytoscape (v3.8.2)	https://cytoscape.org	RRID:SCR_003032
Zeiss Zen Microscopy Software (v3.9)	https://www.zeiss.com/microscopy/en/products/software/zeiss-zen.html	RRID:SCR_013672
Imaris Software (v10.2.0).	https://www.bitplane.com/imaris/imaris	RRID:SCR_007370
Fiji (v1.54)	https://fiji.sc	RRID:SCR_002285
Manchester PLF (MPLF) webtool	https://www.manchesterproteome.manchester.ac.uk/#/MPLF	N/A
GraphPad Prism (v10)	https://www.graphpad.com/	RRID:SCR_002798
Other		
Mouse Express L-Lysine (^13^C_6_, 99%)	CK Isotopes Ltd	Cat#MLK-LYS-C

### Experimental Model and Study Participant Details

#### Animals

The care and use of all mice in this study was carried out in accordance with the UK Home Office regulations, UK Animals (Scientific Procedures) Act of 1986 under the Home Office Project License’s: P1AE9A736, PP4262564, PP708858 and LO45CA465. Mice were maintained in 12-h light/12-h dark cycle at 20°C–22°C, humidity of 40–50%, with *ad libitum* access to water. Mice were fed “light diet” (standard rodent chow) or “heavy diet”; Mouse Express L-Lysine (^13^C_6_, 99%; MLK-LYS-C) purchased from CK Isotopes Ltd (UK), as per the labeling experimental design. For the pulse-time experiments, wildtype male C57BL/6J mice (Charles River) were employed. For the RNA sequencing experiment and Alport proteomics study, male *Col4a5*^−*/*^ mice were employed. These mice were obtained from the International Mouse Phenotyping Consortium^62^ and the line was generated by deletion of the critical exon 36, resulting in the absence of the collagen-α5(IV) protein, using the published allele map.^63^ *Col4a5*^*-/Y*^ mice were on a C57BL/6 background. Heterozygous females were crossed with wildtype males and hemizygous male offspring were used to study Alport syndrome and wildtype male littermates were used as a control. All mice gained weight in accordance with reference laboratory data for C57BL/6J mice.

#### Study participants

For the Alport cohort, human blood samples were obtained under the Manchester Renal Biobank ethics (16/NW/0119). Alport patients were invited to clinic at Royal Manchester Children’s Hospital between the study period 02/11/2022–21/11/2023 and informed consent and assent, where applicable, were obtained. For enrollment into the study, a confirmed pathogenic collagen IV variant was required. Alport patient’s provided samples at their first clinic visit (baseline, *n* = 38) and 6 months later (follow-up, *n* = 30). Serum from healthy children (*n* = 30) was purchased from Discovery Life Sciences (Los Osos, CA, USA). For study participant characteristics, see Figure S8B.

### Method Details

#### Preparation of tissues

Mice were sacrificed by cervical neck dislocation and kidneys were immediately dissected. The surrounding fat, adrenal glands and renal capsule were removed, and kidneys used for RNA sequencing and proteomic experiments were snap frozen and stored at −80°C. Kidneys used for immunofluorescence imaging were embedded in OCT compound and stored at −80°C until cryosectioning.

#### Mouse urine collection and urinary albumin excretion

To assess urinary albumin excretion, spot urine samples were collected from wildtype and Alport mice at 8-, 12-, and 16-week of age (*n* = 5 per condition and time-point) and stored at −80°C. The urinary albumin-to-creatinine ratio (ACR, mg/g) was quantified using a Mouse Albumin ELISA Quantification Set (FORTIS Life Sciences) and Creatinine Kit (Bio-Techne R&D Systems) according to the manufacturer’s instructions.

#### Collection of human and mouse blood samples

For mice, blood (400μL) was collected from wildtype and Alport mice using cardiac puncture following schedule 1 sacrifice. For serum, whole blood was allowed to clot for 30 min at room temperature, then centrifuged (1300 x g) for 15 min at 4°C and serum (*n* = 9 wildtype, *n* = 11 Alport) transferred into clean polypropylene transfer tubes. For plasma, whole blood was added to a tube containing 10μL of EDTA (0.5M, pH 8.0) (Sigma), mixed thoroughly and immediately centrifuged (2000 x g) for 10 min at 4°C and plasma (*n* = 6 wildtype, *n* = 7 Alport) was transferred to clean polypropylene transfer tubes. For patients with Alport syndrome, whole blood (5 mL) was collected into an SST (serum separator tube) following an 8-h fast and allowed to clot for 30 min at room temperature. Samples were centrifuged (2000 x g) for 15 min at 4°C within 1 h of collection and serum was transferred into a clean polypropylene transfer tube. All samples were immediately stored at −80°C until transportation to Nordic Bioscience (Denmark) for analysis. Serum creatinine and urine protein creatinine ratio were also measured at the clinic visit, in keeping with standard operating procedures of the biochemistry laboratory at Royal Manchester Children’s Hospital.

#### Histology

Kidney tissue was obtained from wildtype and Alport mice at 4-, 8-, 12-, 16-, 20-, and 24-week of age (*n* = 3 mice per genotype per timepoint). Formalin-fixed tissues were dehydrated and cleared before embedding into paraffin wax blocks. Tissue blocks were sectioned at 3 μm thickness and for each mouse, a minimum of three sections spaced 100 μm apart were cut and stained using a Leica XL ST5010 Autostainer. Sections were incubated at 65°C for 10 min and dewaxed by immersion in xylene (2 × 2 min), followed by 100% ethanol (1 × 2 min, 1 × 1 min), 70% ethanol (1 × 30 s) and then rinsed in running tap water. Sections were stained with picrosirius red (PSR) solution (1 g Direct Red 80 dissolved in 1000 mL of 1.3% picric acid) for 1 h at room temperature. Slides were rinsed in 1% acetic acid (5 s), then dehydrated in 95% ethanol (1 min) and 100% ethanol (1 min), cleared in xylene (2 ×with excitation at 488 or 594 nm. Glomeruli 2 min), and mounted using Pertex mounting medium. Whole-kidney sections were imaged and stitched using the Olympus VS200 Slide-scanner (Olympus, Japan) with a 20X objective. Quantification of PSR staining was performed using QuPath (version 0.6.0).^64^ Brightfield images were used to manually outline regions of interest (ROIs) around the renal cortex by a blinded researcher. Non-specific perivascular and capsular staining were excluded. The ROIs were then applied to the corresponding polarized light images to quantify collagen signal. For each mouse, three technical sections were analyzed and averaged. A single pixel classifier was developed and applied across all slides using the same threshold parameters. Under polarised light, thick collagen fibers were quantified using red birefringence, while thin collagen fibers were quantified using green birefringence and the percentage area of red and green signal within the ROI was calculated.

#### Immunofluorescence and imaging

Frozen kidney sections with a thickness of 10 μm were obtained using a Leica CM1950 cryostat. Coronal kidney sections were washed in PBS to remove the OCT compound, air-dried and fixed with cold acetone for 10 min. Acetone-fixed sections were blocked with blocking buffer (0.2% fish skin gelatin, 0.05% Triton X-100/PBS) for 1 h at 4°C and then incubated with primary antibody diluted in blocking buffer overnight at 4°C. After washing with 0.2% Tween 20 in PBS, sections were incubated with secondary antibodies for 3 h at room temperature. Following washing in PBS, nuclei were stained with Hoechst 33258 and mounted with ProLong Gold Antifade mountant (P36934; Invitrogen). Images were acquired at 50X magnification with a Zeiss Cell Discoverer 7 inverted confocal microscope (CD7) using a Plan-Apochromat/1.2 50X objective with a 0.5X Tubelens and excitation at 488 or 594 nm. When acquiring 3D optical stacks, the confocal software was used to determine the optimal number of Z sections. Images were processed using Zen (version 3.9) and Imaris software (version 10.2.0). Images for quantification were collected on an inverted Leica SP8 confocal microscope using an HC PL APO 63x/1×, 40× objective with excitation at 488 or 594 nm. Glomeruli were manually segmented, and mean fluorescence intensity calculations were automated in ImageJ (v1.54).

#### *In situ* detection of collagenase activity

Frozen kidney sections from Alport (*n* = 5) and wildtype (*n* = 5) mice, with a thickness of 10 μm, were obtained using a Leica CM1950 cryostat. Coronal kidney sections were washed in 1X Reaction Buffer from EnzChek Gelatinase/Collagenase Assay Kit (E12055; Invitrogen). Subsequently, 1 mg/mL DQ collagen type I (D-12060; Invitrogen) was applied to the sections. After overnight incubation in the dark at room temperature, kidney sections were washed once with PBS and fixed with cold acetone, followed by immunofluorescence staining as described above. Images were acquired using a Leica SP8 confocal microscope equipped with a 20X objective lens. Using dual-channel images (DQ collagen type I/anti-perlecan immunofluorescence staining), sixty-three and eighty glomeruli were randomly chosen from control (10, 6, 13, 23 and 11 glomeruli) and Alport (20, 11, 14, 15 and 20 glomeruli) kidneys, respectively. The regions of interest (ROIs) of individual glomerulus were determined on the perlecan images using ImageJ (v1.54). Mean intensity of DQ-Col I signal in the ROIs were quantified and statistically analyzed.

#### RNA extraction

Total RNA was extracted from 30 mg of kidney tissue using TRIzol reagent (Invitrogen). In brief, 1 mL of TRIzol was added to minced kidney tissue and homogenized with a micro pestle. Following phase separation with 0.2 mL of chloroform, the aqueous phase was mixed with 0.5 mL of isopropanol and centrifuged for 10 min at 12,000 x g at 4°C. RNA precipitate was washed with 75% ethanol. RNA in 100 μL of RNase-free H_2_O was cleaned up using RNeasy mini column (QIAGEN) as per manufacturers protocol. RNA was eluted in nuclease-free water and concentration measured using a NanoDrop 2000 (Thermo Fisher Scientific). The quality of all resuspended mRNA was measured using a Qubit Fluorometer (Thermo Fisher Scientific) and RNA integrity measured using a 2200 TapeStation (Agilent Technologies) to ensure good quality for downstream analysis.

#### Bulk RNA sequencing and data analysis

RNA was checked for quality and prepared for sequencing in the Genomic Technologies Core Facility in Manchester. Unmapped paired-end sequences from an Illumina HiSeq4000/NovaSeq 6000 sequencer were quality controlled by inhouse software using several open-source tools (https://github.com/fls-bioinformatics-core/auto_process_ngs). Sequence adapters were removed, and reads were quality trimmed using Trimmomatic (v0.39).^65^ The reads were mapped against the reference mouse genome (mm10/ GRCm38) and counts per gene were calculated using annotation from GENCODE M32 (http://www.gencodegenes.org/) using STAR (v2.7.7a).^66^ Normalization, Principal Components Analysis, and differential expression was calculated with DESeq2 (v1.40.2).^67^ Adjusted *p*-values were corrected for multiple testing (Benjamini and Hochberg method). Gene ontology enrichment was studied using clusterProfiler (v4.8.3)^68^ and Enrichr (v3.2).^69^ Gene enrichment was studied using ReactomePA (v1.44.0)^70^ and Ingenuity Pathway Analysis (QIAGEN).^71^

#### Pulsed ^13^C-Lys labeling experimental design

For the pulse-time experiments, wildtype mice were pulse-labelled with ^13^C-Lys (heavy diet) for 1-, 2, 4- or 8-week (*n* = 3 per time-point) and were sacrificed at 22 weeks for tissue harvesting (see Figure 2B). For the Alport syndrome study, mice were fed either 4- or 8-week pulses (*n* = 4 per time-point per genotype) of ^13^C-Lys diet and were sacrificed aged 16 weeks for tissue harvesting (see Figure 3A). Following addition of heavy-isotope variants of amino acids in diet, “light” versions of proteins are replaced with chemically indistinguishable “heavy” versions over time (see Figure 2A). The purpose of pulse-labelling for different periods was to monitor label incorporation over time to allow protein half-life calculation using a recognized mass spectrometry workflow (MATLAB).^35^

#### Matrix-enrichment protocol for proteomic experiments

Frozen-thawed kidneys were placed in a glass dish, cut into small pieces (<1 mm^3^) and washed three times with ice-cold PBS. To solubilize the kidney, 200 μL of SL-DOC buffer (1.1% (vol/vol) sodium laurate; 0.3% (vol/vol) sodium deoxycholate; 25 mM ammonium bicarbonate; 0.5 mM dithiothreitol (DTT); protease and phosphate inhibitors as per manufacturer’s guidance (Sigma); pH 7) was added to the kidney tissue along with six 1.6 mm steel beads. Samples were homogenized at maximum speed for 5 min at 4°C using a Bullet Blender Tissue Homogenizer (Next Advance, New York, USA). Samples were alkylated with iodoacetamide (IAA) (final concentration, 45 mM), before quenching with DTT. Following centrifugation, supernatants were collected and transferred to LoBind Protein tubes. This sample was designated soluble fraction 1 (F1; cellular compartment). Remaining pellets were further solubilized using 75 μL of High Salt Buffer (3 M sodium chloride; 25 mM DTT; 25 mM ammonium bicarbonate, protease and phosphate inhibitors as per manufacturer’s guidance; pH 7) and further homogenized using a bullet blender at maximum speed for an additional 5 min. Following centrifugation, remaining pellets were sonicated in 300 μL of Lysis buffer (5% sodium dodecyl sulfate (Sigma); 50 mM triethylammonium bicarbonate (TEAB); pH 7), supplemented with protease and phosphatase inhibitors, using a Covaris LE220+ Sonicator (40W program; 100 cycle per burst; duty factor 40%; peak incident power 500W). The protein samples were reduced with DTT (final concentration, 5 mM), followed by alkylation and quenching as above. To generate the matrix-enriched fraction 2 (F2; matrix compartment), the supernatants from the High Salt and Lysis buffer extractions were pooled. Protein concentrations in F1 and F2 were determined by BCA protein assay kit (Pierce Rapid Gold BCA Protein Assay Kit, Thermo Fisher Scientific).

#### Preparation of samples for mass spectrometry analysis

50 μg of protein from each sample was prepared in 5% SDS to a total volume of 50 μL and then acidified using 5 μL of 12% H_3_PO_4_. 350 μL binding buffer (90% methanol, 10% distilled water; 100 mM TEAB) was added and the samples loaded onto an S-Trap plate (ProtiFi S-Trap micro-plate) according to the manufacturer’s protocol. Bound proteins were washed in binding buffer and methyl *tert*-butyl ether (MTBE) and then digested overnight at 37°C with 5 μg of trypsin in Digestion buffer (50 mM TEAB; 0.1% formic acid, FA; at pH 8.5). Peptides were eluted from the column in 65 μL Digestion buffer, 65 μL 0.1% FA in distilled water, and finally with 30 μL 0.1% FA, 30% acetonitrile in water. Peptides were desalted using Oligo R3 resin beads (Thermo Fisher), according to the manufacturer’s protocol, in a 96-well, 0.2 μm polyvinylidene fluoride filter plate AB gene). The immobilized peptides were washed twice with 0.1% FA prior to elution with 0.1% FA with 30% ACN, and lyophilization.

#### Mass spectrometry

Peptides were resuspended in 10 μL 0.1% FA in 5% ACN. Liquid chromatography (LC) separation was performed on a Thermo RSLC system consisting of an NCP3200RS nano pump, WPS3000TPS autosampler and TCC3000RS column oven configured with buffer A as 0.1% (vol/vol) FA in water and buffer B as 0.1% (vol/vol) DA in ACN. The analytical column (Waters nanoEase M/Z Peptide CSH C18 Column, 13000C5;, 1.7μm, 75μm × 250mm) was kept at 35°C and at a flow rate of 300 nL/min for 8 min. The injection valve was set to load before a separation consisting of a 105-min multistage gradient ranging from 2 to 65% of buffer B. The LC system was coupled to a Thermo Exploris 480 mass spectrometry system via a Thermo Nanospray Flex ion source. The nanospray voltage was set at 1900V and the ion transfer tube temperature set to 275°C. Data was acquired in a data-dependent manner using a fixed cycle time of 2 s, an expected peak width of 15 s and a default charge state of +2. Full mass spectra were acquired in positive mode over a scan range of 300 to 1750Th, with a resolution of 120000, a normalized automatic gain control (AGC) target of 300% and a maximum fill time of 25 ms for a single microscan. Fragmentation data was obtained from signals with a charge state of +2 or +3 with an intensity over 5000 and they were dynamically excluded from further analysis for a period of 15 s after a single acquisition within a 10-ppm window. Fragmentation spectra were acquired with a resolution of 15000, a normalized collision energy of 30%, an AGC target of 300%, a first mass of 110Th, and a maximum fill time of 25 ms for a single microscan. All data was collected in profile mode.

#### Proteomics data analysis

Mass spectrometry analysis was carried out using a SILAC 1plex (Lys6) method in Proteome Discoverer (Thermo Fisher Scientific, v3.2). Carbamidomethylation of cysteine was selected as a fixed modification and variable modifications included oxidation of methionine, hydroxylation of proline and phosphorylation of threonine, serine, and tyrosine. During specific analysis of post-translational modifications, MS search parameters were widened to include semi-tryptic peptides, allowing detection of degraded proteins. A maximum of 2 missed cleavages per peptide was allowed. The minimum precursor mass was set to 350 Da with a maximum of 5000 Da. Precursor mass tolerance was set to 10 ppm, fragment mass tolerance was 0.02 Da and minimum peptide length was 6. Peptides were searched against the mouse Swiss-Prot database with additional TreEMBL matrix protein sequences (unreviewed) using the SEQUEST HT search tool. Identified proteins were required to have a minimum false discovery rate (FDR) of 1% and at least 2 unique peptides. Known contaminants were removed. Abundance was calculated from the MS1 ion current peaks and combined for both heavy and light channels. Values were exported to Perseus (v2.0.11.0) for further analysis, proteins were filtered for those with at least 3 calculated abundances, normalized by median subtraction (either to all proteins or, for F2, to matrisome proteins) and then missing data was imputed based on standard deviation. Heavy-to-light ratios were determined using the abundance of a protein detected in the heavy channel, containing ^13^C-lysine, compared to the light channel. Trypsin was selected as the digestion enzyme as it is the standard protease for proteomics workflows, generating peptides of optimal size for LC-MS/MS analysis. As only lysine residues were isotopically labeled, tryptic peptides terminating in arginine do not incorporate the heavy label and are excluded from heavy-to-light ratio quantification, reducing the available peptide pool for turnover analysis to approximately 50% of all tryptic peptides. This is a recognised feature of lysine-only SILAC labeling and did not affect the validity of turnover measurements, which were based on multiple quantified lysine-terminating peptides per protein across biological replicates. Heavy-to-light ratios were converted to half-lives using published mass spectrometry workflow (MATLAB) developed by Alevra et al.^35^ A minimum of 5 data points (pulse-times or biological repeats) were required for half-life calculation from the pulse-time experiment or 8 data points from the Alport experiment, for which data was fed to the pulse-time experiment model. Default parameters were used for modeling lysine pools and model fit was based on adult F1 samples. Protein and peptide lists were filtered for matrix proteins using R code (v4.1.2) based on their presence in MatrisomeDB^33^ and bmBASE.^15^ Protein interaction network analysis was performed using STRING software (v11.5).^34^ interactions were filtered for high confidence (minimum interaction score of 0.7), and networks were visualized using Cytoscape (v3.8.2).^72^

#### Peptide location fingerprinting

PLF was performed using our predeveloped Manchester PLF (MPLF) webtool (https://www.manchesterproteome.manchester.ac.uk/#/MPLF.),^29^ as previously described.^29–31^ Peptide lists were imported into the webtool where proteins were automatically segmented into 50 aa-sized segments and peptide sequences with their associated exclusive spectrum counts were mapped and summed within each. To measure regional differences in peptide yield across protein structures that are independent from changes in whole protein abundance, summed peptide counts per segment were median normalized based on their corresponding individual protein total spectrum count. Regional Alport-associated differences in peptide yield across protein structures were visualized by subtracting the average, normalized peptide counts per segment in the Alport group from the wildtype group and dividing by the segment length. Normalized peptide counts per segment were statistically compared between Alport and wildtype groups using a Bonferroni-corrected, unpaired repeated measure ANOVA. For more information on PLF and its application, please refer to the original MPLF development publication.^29^

#### Peptide sequence turnover analysis

Proteins were segmented into 100aa sized segments, peptides were then matched to these segments, grouped, and average heavy-to-light ratios were calculated. Fold changes were calculated for each segment compared to the average turnover of the protein in order to focus on areas of interest along the protein sequence. To illustrate variation in ^13^C-lysine incorporation across protein sequences, representative MS1 spectra are provided for peptides from within and outside regions of structural interest for COL1A1 and LAMA5 (Figure S10).

#### Detection of circulating matrix fragments

The ECM biomarkers C1SIG (nordicC1SIG), LG1M (nordicLG1M), NIC (nordicNIC), and C4M (nordicC4M) were quantified in serum from healthy children and children with Alport syndrome. The ECM biomarkers C1M (nordicC1M) and rCTX-III (nordicrCTX-III) were quantified in mouse serum or plasma, respectively. The biomarkers were measured using competitive enzyme-linked immunosorbent assays (ELISAs) developed and produced at Nordic Bioscience (Herlev, Denmark) according to the manufacturer’s instructions. The C1SIG assay reflects type I collagen degradation mediated by MMP-2 and MMP-9 detecting sequence RTGDAGPVGP located in the alpha-1 chain (unpublished). The LG1M assay measures laminin degradation mediated by MMP-9 detecting the sequence LNRKYEQAKN corresponding to the cleavage site in the laminin gamma 1 chain.^45^ The NIC assay measures Cathepsin S mediated nidogen-1 degradation detecting sequence VEKTRCQHERE.^47^ C4M reflects MMP-2, -9, and -12-mediated degradation of type IV collagen detecting sequence ILGHVPGMLL positioned in αlpha-1 (IV) chain.^46^ The C1M assay measures type 1 collagen degradation mediated by MMP-2, -9, and -13 detecting the sequence GSPGKDGVRG located in the alpha-1 chain of type 1 collagen.^39^ The rCTX-III assay measures type III collagen degradation mediated by MMP-9 and -13 detecting the sequence KSGGFSPYYG which reflects a neoepitope cleaved from the C-terminal telopeptide of type III collagen.^40^ The inter- and intraassay coefficients of variation (CV) are >15% and 10%, respectively for all assays. Samples below the lower limit of quantification (LLOQ) were assigned the value of LLOQ.

#### Quantification and Statistical Analysis

Data was tested for normality where appropriate using the D’Agostino and Pearson test. For non-parametric data, the statistical tests employed were Mann-Whitney (when comparing two conditions) and Kruskal-Wallis (when comparing more than two conditions) tests. FDR corrections were applied when multiple comparisons were made. Where normally distributed data were detected, t-tests or ANOVAs were employed, depending on the number of comparisons being made. For the two-level factor analysis of heavy-to-light ratios in Figure 5A, data was log_10_ transformed, residuals were examined for normality and as these were normally distributed, a two-way ANOVA was used for this analysis. Data are presented as mean ± SEM unless otherwise indicated in figure legends. Sample number (n) indicates the number of independent biological samples in each experiment. Sample numbers and technical replicates are indicated in figures and figure legends or methods section above. Data were analyzed as described in the legends and all data analysis was not blinded. Differences in means were considered statistically significant at *p* < 0.05. Significance levels are: *p < 0.05; **p < 0.01; ***p < 0.001; ****p < 0.0001. Actual *p*-values are shown where appropriate. Statistical analyses were performed using the GraphPad Prism software (version 10).

## Supplementary Material

Supplemental information

Supplementary Table S1

Supplementary Table S2

## In brief

The temporal and spatial dynamics of kidney basement membrane remodeling during disease are largely unknown. Using metabolic labeling combined with proteomics, Preston et al. uncover accelerated matrix turnover and structural destabilization in Alport syndrome, linking protease activity to circulating collagen and laminin fragments with biomarker potential for earlier disease detection.

## Highlights

- Kidney basement membrane turnover accelerates in Alport syndrome
- Protease pathways are activated in Alport kidneys
- Peptide location fingerprinting predicts disease-specific collagen fragmentation
- Circulating matrix fragments are detected in children with Alport variants
