# Supplementary material for: Accelerated basement membrane remodeling and serum matrix fragments as biomarkers of fibrosis in Alport syndrome
Source: Cell Rep. Author manuscript; Available in PMC 2026 Oct 9. (PMC7619522; doi:10.1016/j.celrep.2026.117356)
Supplement: Supplementary file 1 [file EMS219137-supplement-Supplemental_information.pdf]

Cell Reports, Volume *45*

Supplemental information

Accelerated basement membrane remodeling and serum matrix fragments as biomarkers of fibrosis in Alport syndrome

Rebecca Preston, Anna Hoyle, Emily Williams, Alana Stevenson Harris, Ko Tsutsui, Chloe Williams, Catherine Park, David Spiller, Leo Zeef, Solveig Skovlund Groen, Federica Genovese, Joan Chang, Qing-Jun Meng, Joe Swift, Alexander Eckersley, and Rachel Lennon

Accelerated basement membrane remodeling and serum matrix fragments as biomarkers of fibrosis in Alport syndrome

Rebecca Preston[1,2], Anna Hoyle[3], Emily Williams[1,2], Alana Stevenson Harris[1,2], Ko Tsutsui[1,2], Chloe Williams[1,2], Catherine Park[4], David Spiller[4], Leo Zeef[5], Solveig Skovlund Groen[6], Federica Genovese[6], Joan Chang[2,7], Qing-Jun Meng[1,2], Joe Swift[1,2], Alexander Eckersley[2,8], Rachel Lennon[1,2,9,10,11,*]

1. *Division of Cell Matrix Biology & Regenerative Medicine, School of Biological Sciences, Faculty of Biology Medicine and Health, The University of Manchester, Manchester Academic Health Science Centre, Manchester, M13 9PT, UK.*
2. *Manchester Cell-Matrix Centre, School of Biological Sciences, Faculty of Biology Medicine and Health, The University of Manchester, Manchester Academic Health Science Centre, Manchester, M13 9PT, UK.*
3. *Kennedy Institute of Rheumatology, The University of Oxford, Oxford, OX3 7FY, UK*
4. *Bioimaging Core Facility, School of Biological Sciences, Faculty of Biology Medicine and Health, The University of Manchester, Manchester, M13 9PT, UK.*
5. *Bioinformatics Core Facility, School of Biological Sciences, Faculty of Biology Medicine and Health, The University of Manchester, Manchester, M13 9PT, UK.*
6. *Cardiovascular and Renal Research, Nordic Bioscience, Herlev Hovedgade 205, 2730 Herlev, Denmark.*
7. *Division of Molecular & Cellular Function, School of Biological Sciences, Faculty of Biology Medicine and Health, The University of Manchester, Manchester Academic Health Science Centre, Manchester, M13 9PT, UK.*
8. *Division of Musculoskeletal & Dermatological Sciences, School of Biological Sciences, Faculty of Biology Medicine and Health, The University of Manchester, Manchester Academic Health Science Centre, Manchester, M13 9PT, UK.*
9. *Department of Paediatric Nephrology, Royal Manchester Children's Hospital, Manchester University Hospitals NHS Foundation Trust, Manchester Academic Health Science Centre, Manchester, M13 9WL, UK.*
10. *Senior author*
11. *Lead contact*

*Correspondence: Rachel.lennon@manchester.ac.uk

CONTENTS

S. Figure 1	page 3
S. Figure 2	page 5
S. Figure 3	page 7
S. Figure 4	page 9
S. Figure 5	page 10
S. Figure 6	page 12
S. Figure 7	page 13
S. Figure 8	page 15
S. Figure 9	page 17
S. Figure 10	page 19

Supplementary Figure 1

A Progressive albuminuria in Alport mice

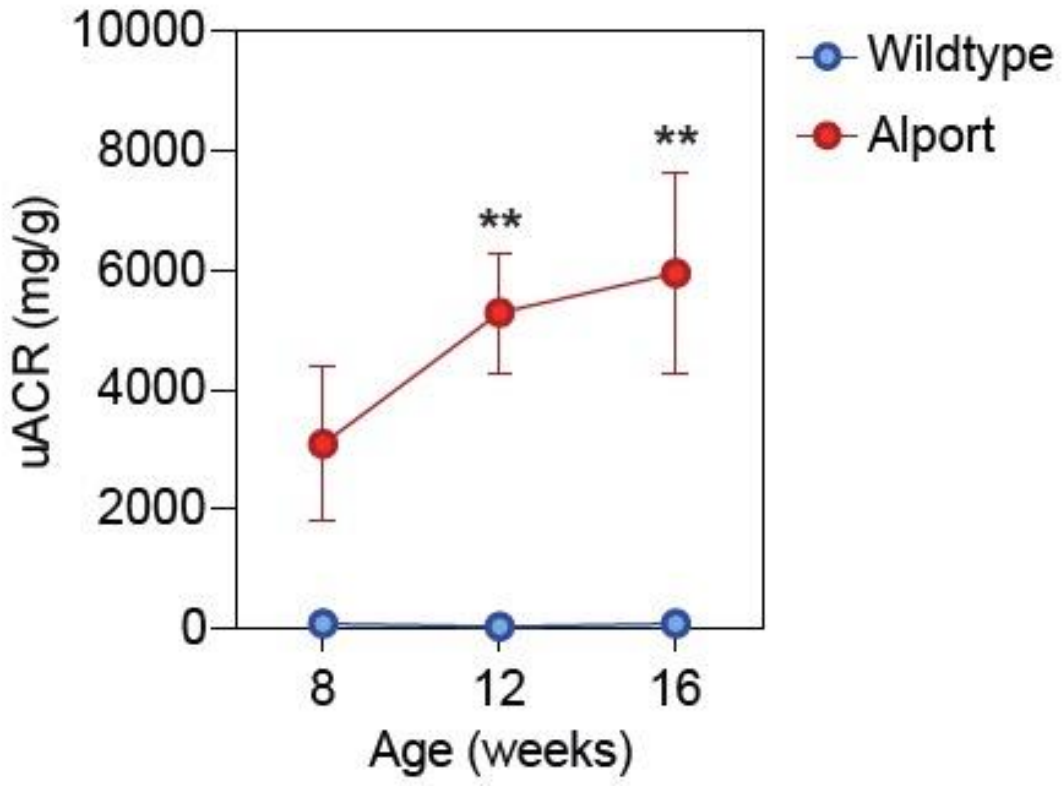

C Quantification of picrosirius red staining in kidney tissue sections

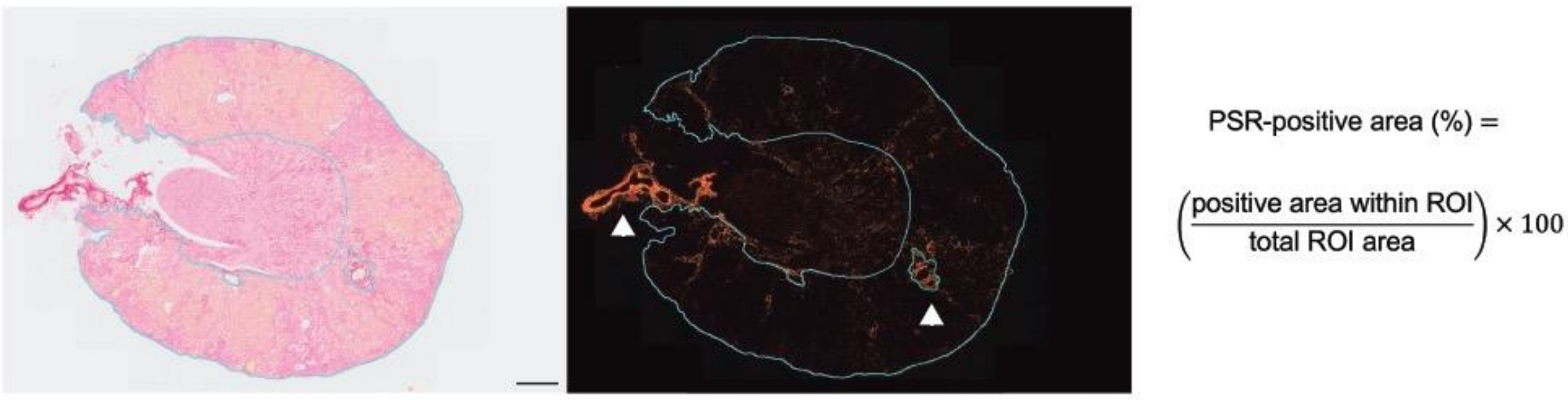

B Picrosirius red staining demonstrates progression of kidney fibrosis in Alport mice

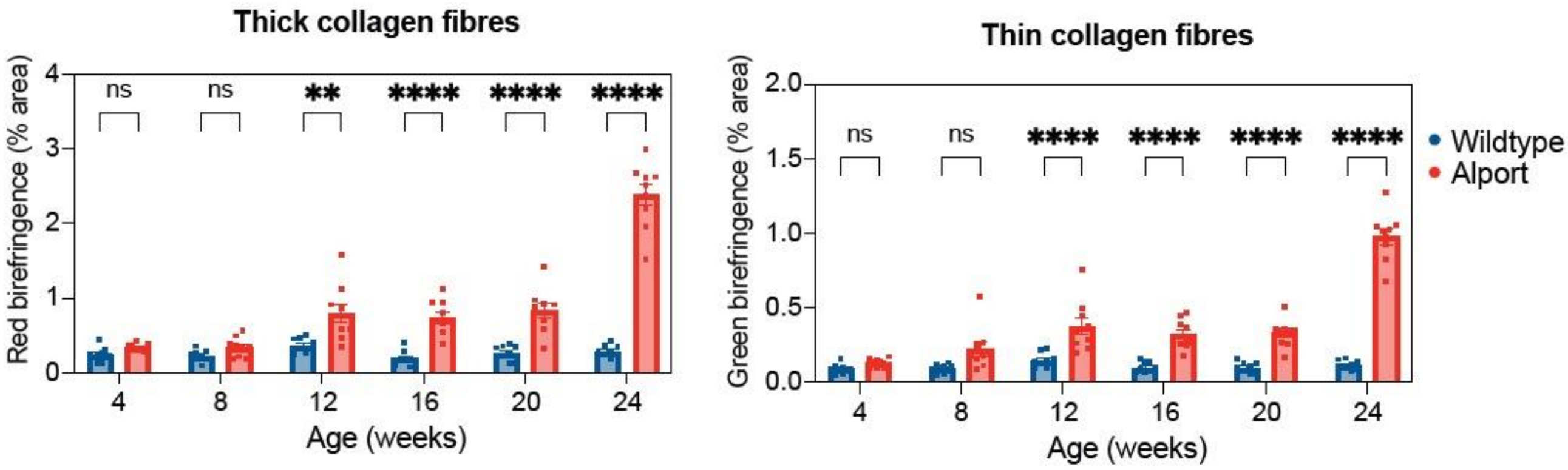

Supplementary Figure 1: Characterization of *Col4a5*$^{-/}$ mice. A) Urine albumin creatinine ratios (mg/g) in wildtype (n=5, shown in blue) and Alport (n=5, shown in red) mice at 8-, 12-, and 16-weeks, demonstrating progressive albuminuria in Alport mice. **p=0.0082 and **p=0.0028 by two-way ANOVA with multiple comparisons. Bars show mean±SEM. **B**) Technique for quantifying picrosirius red staining from kidney tissue sections. Kidney cortex was manually outlined on brightfield images (shown in cyan), non-specific perivascular and capsular staining and artefact was excluded (shown with white arrowhead), and regions of interest were applied to corresponding polarised light images to quantify percentage area of red and green birefringence. **C**) Quantification of kidney fibrosis progression in Alport mice, compared to wildtype controls, using picrosirius red staining demonstrating significantly increased thick (red birefringence; left panel) and thin (green birefringence; right panel), evident from 12-weeks. **p=0.0036 and ****p=<0.0001 by two-way ANOVA with multiple comparisons. Bars show mean±SEM and dots represent individual measurements (n=3 biological and n=3 technical replicates per time-point).

Supplementary Figure 2

A Picrosirius red staining demonstrating progressive fibrosis in Alport kidney

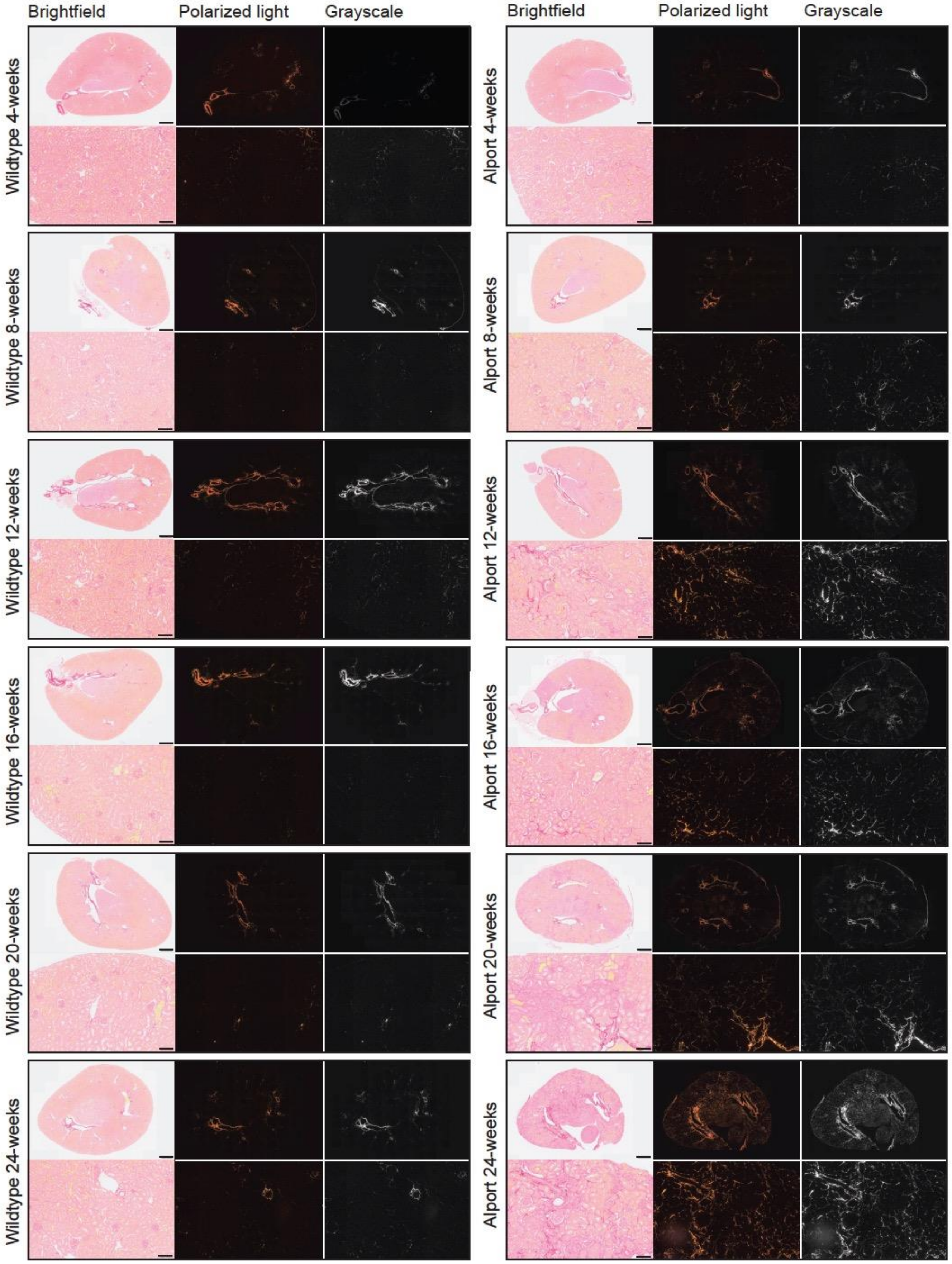

Supplementary Figure 2: Progressive interstitial fibrosis in Alport kidney. A) Representative images of picrosirius red staining in wildtype (left) and Alport (right) kidney at 4-, 8-, 12-, 16-, 20-, and 24-weeks of age. For each time-point, whole kidney sections are shown in the upper panel and magnified region of kidney cortex shown in the lower panel. Each time-point includes brightfield (left), polarized light (middle) and grayscale (right) images. Images acquired at 20X magnification using an Olympus VS200 Slide-scanner. Bars, 500μm for whole kidney sections and bars, 100μm for magnification of kidney cortex.

Supplementary Figure 3

A Significantly upregulated kidney matrix regulators

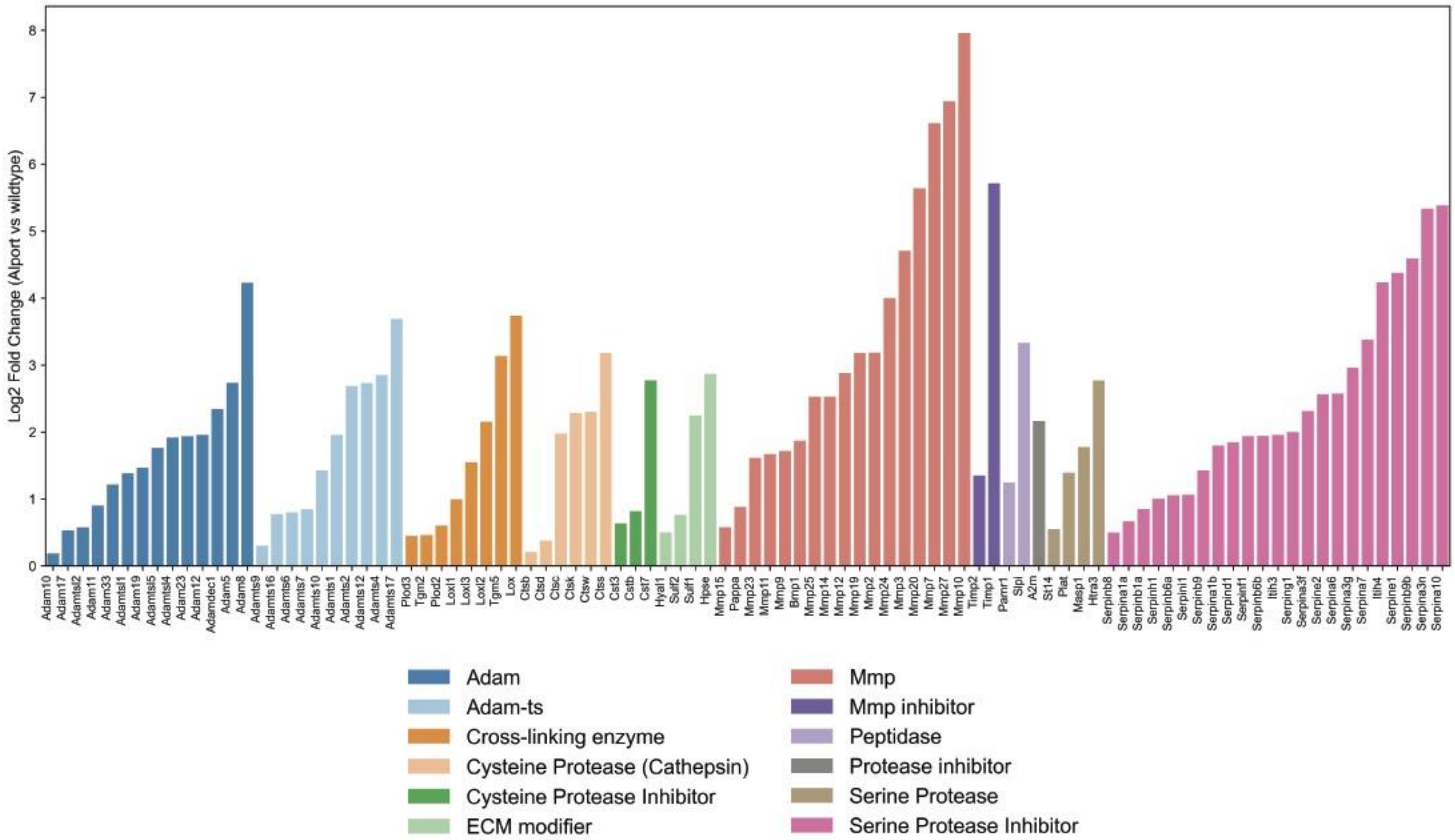

B Top 20 reactome pathways enriched in Alport kidney

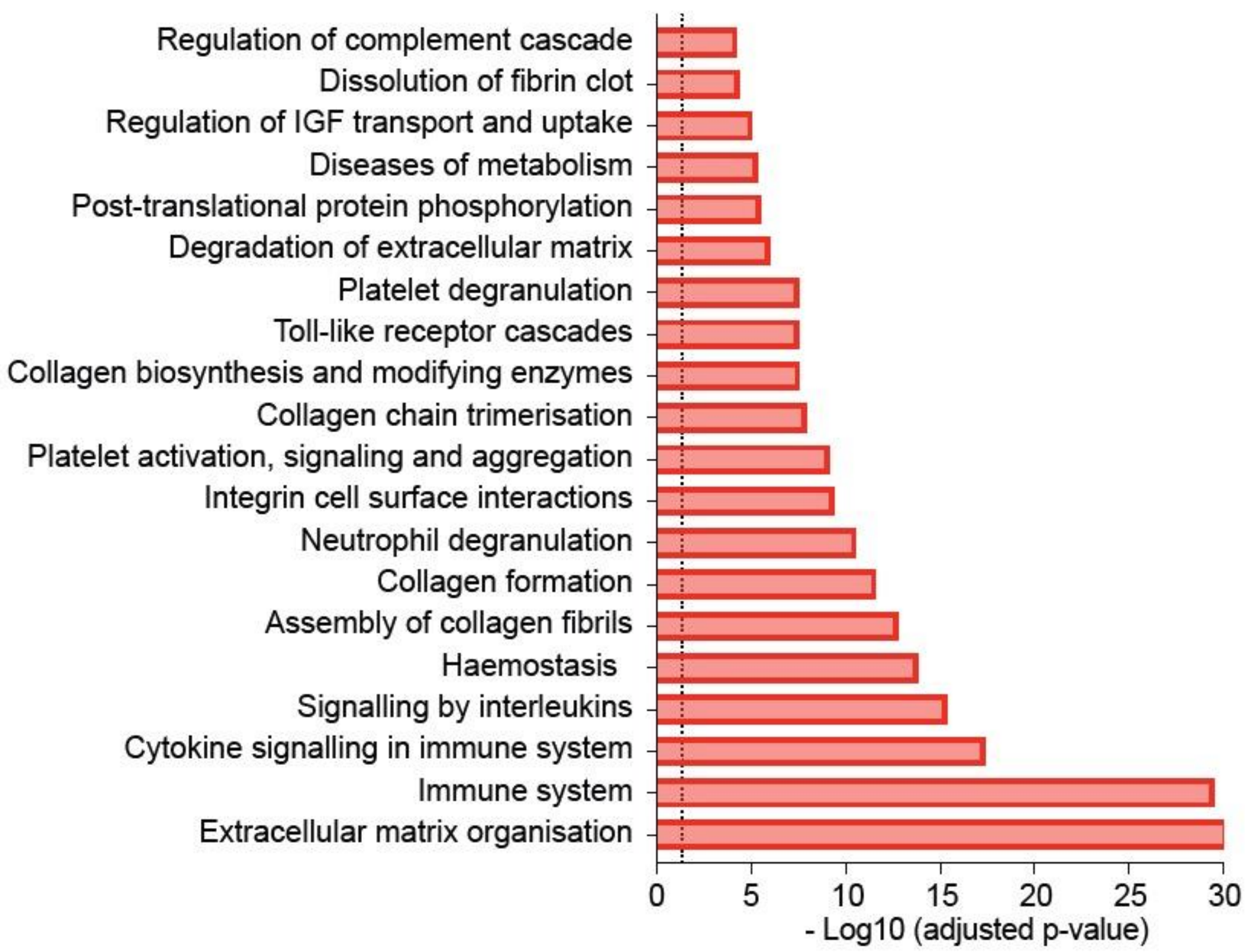

Supplementary Figure 3: Upregulated genes and pathways in Alport kidney. A) Bulk RNA sequencing of Alport versus wildtype kidney demonstrated significant upregulation of 94 kidney matrix regulators important in basement membrane component degradation and collagen cross-linking. **B**) Reactome pathway analysis of the Alport kidney transcriptome, showing the top 20 enriched pathways for genes significantly upregulated in Alport kidney. Black dotted line represents significance threshold (adjusted p-value <0.05). N=3 biological replicates per condition.

Supplementary Figure 4

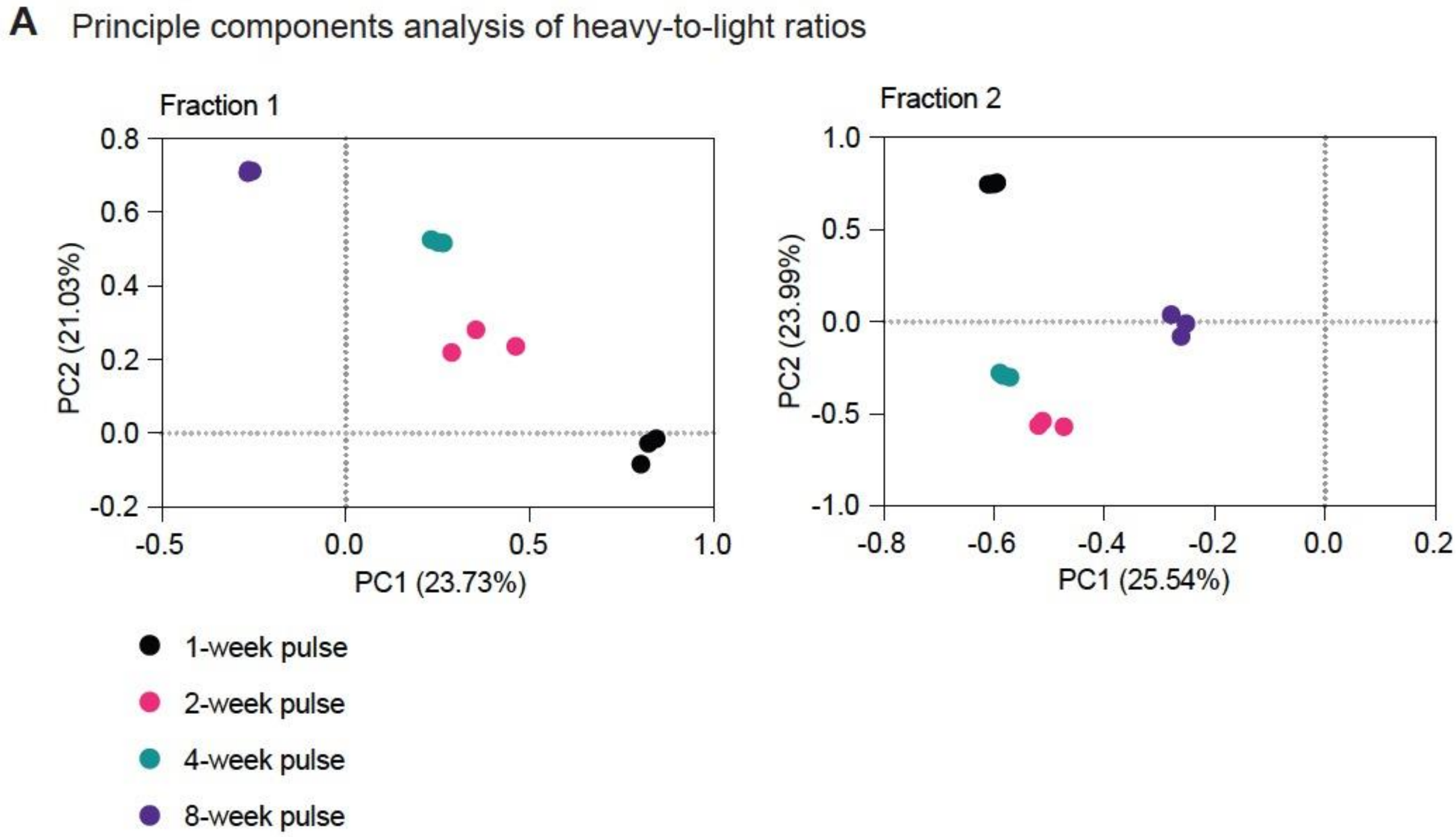

Supplementary Figure 4: Principal Components Analysis. **A)** Principal Components Analysis (PCA) of heavy-to-light ratios in Fraction 1 samples (left panel) and Fraction 2 samples (right panel) from kidneys of mice fed for different pulse-times. N=3 biological replicates per condition.

Supplementary Figure 5

A Principle components analysis of normalized abundance

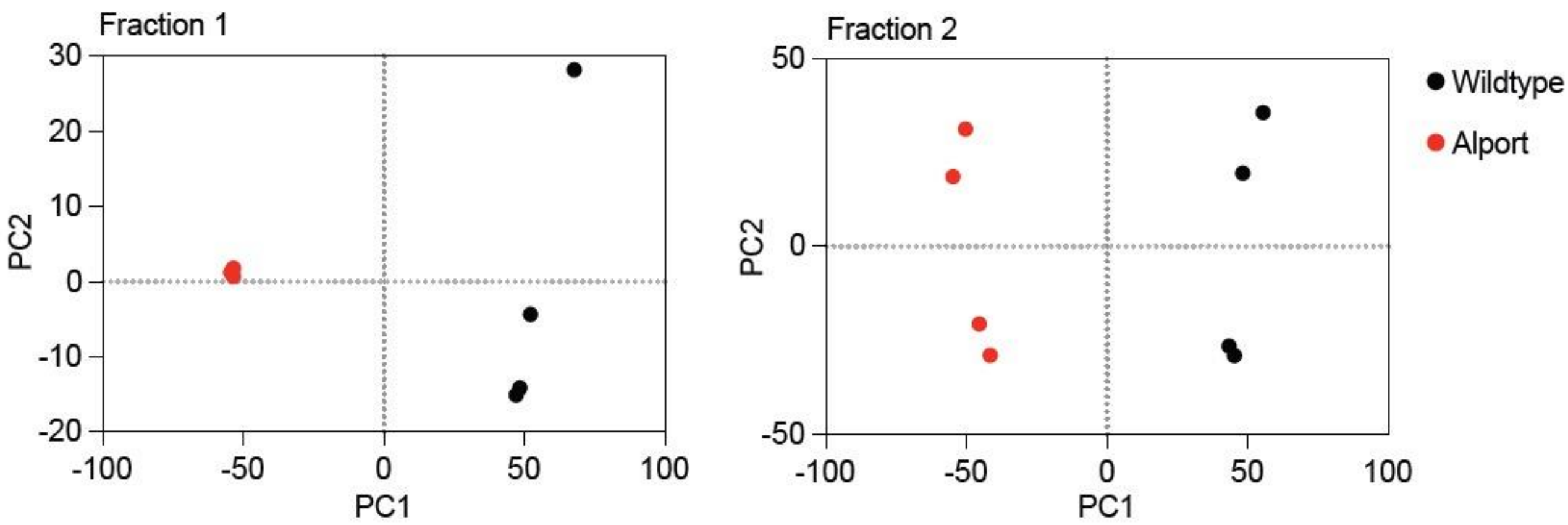

B Differentially expressed proteins in Fraction 1

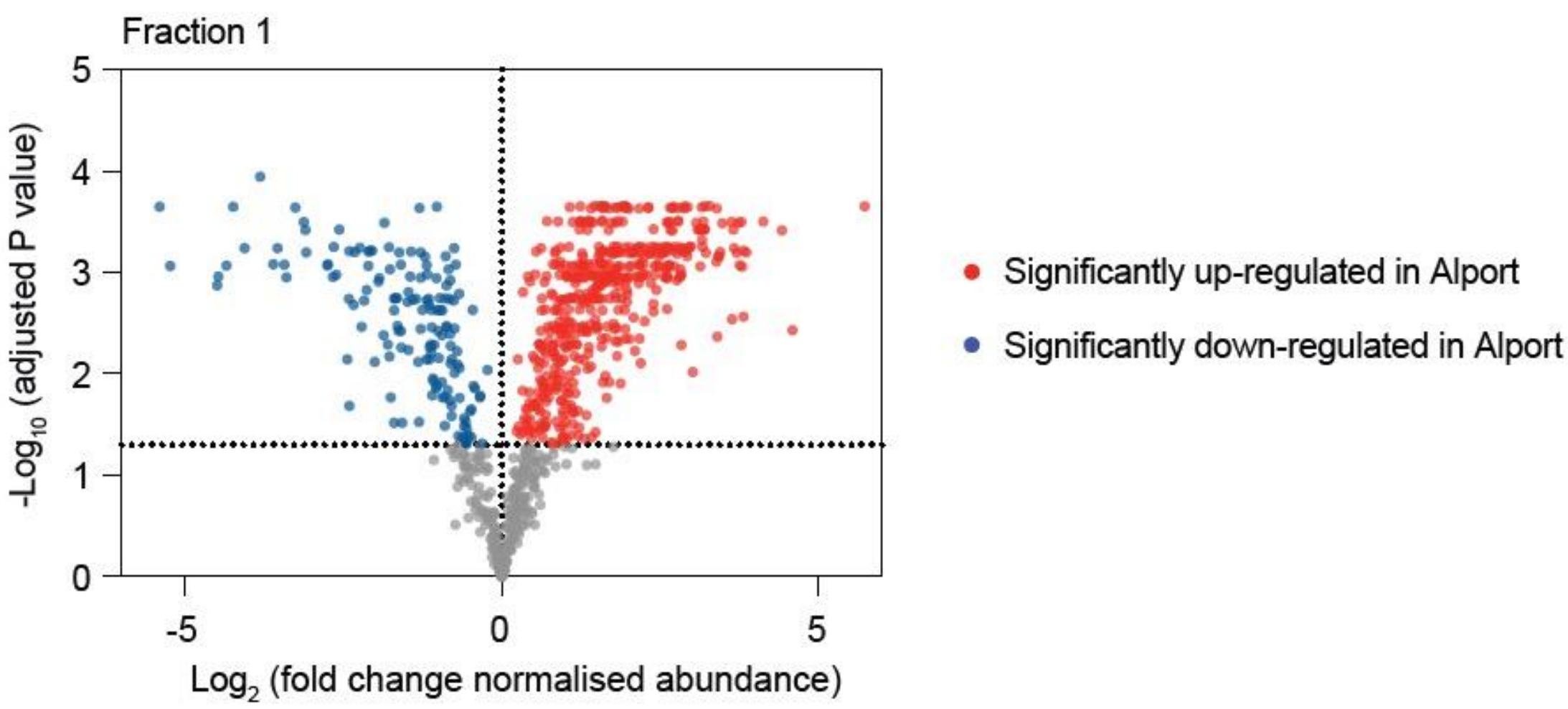

C Reactome pathway enrichment for proteins with increased abundance in Alport kidney

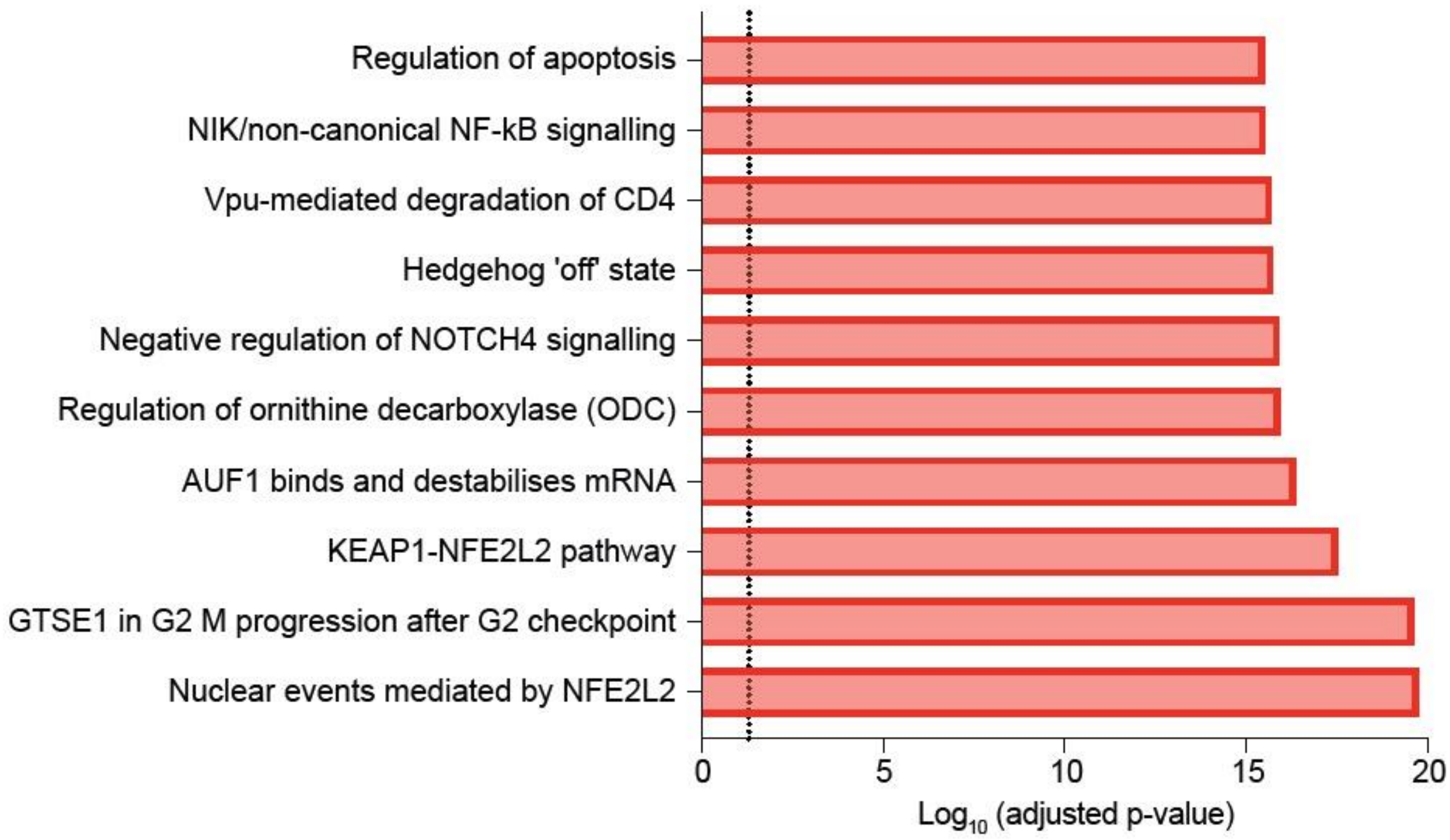

Supplementary Figure 5: Kidney matrix composition in Alport kidney. A) Principal Components Analysis (PCA) of normalized abundance in Fraction 1 samples (left panel) and Fraction 2 samples (right panel) from wildtype (black dots) and Alport (red dots) kidney. N=4 biological replicates per condition. **B**) Volcano plot showing fold-changes in abundance of kidney proteins identified in Fraction 1 from Alport and wildtype kidney. Proteins with significantly increased abundance in Alport kidney are shown in red and proteins with significantly decreased abundance in Alport kidney are shown in blue. Dotted black line represents significance threshold (adjusted p-value <0.05). **C**) Reactome pathway enrichment for cellular proteins detected in Fraction 1 with significantly increased abundance in Alport compared to wildtype mice. Dotted black line represents significance threshold (adjusted p-value <0.05). N=4 biological replicates per condition for all analysis.

Supplementary Figure 6

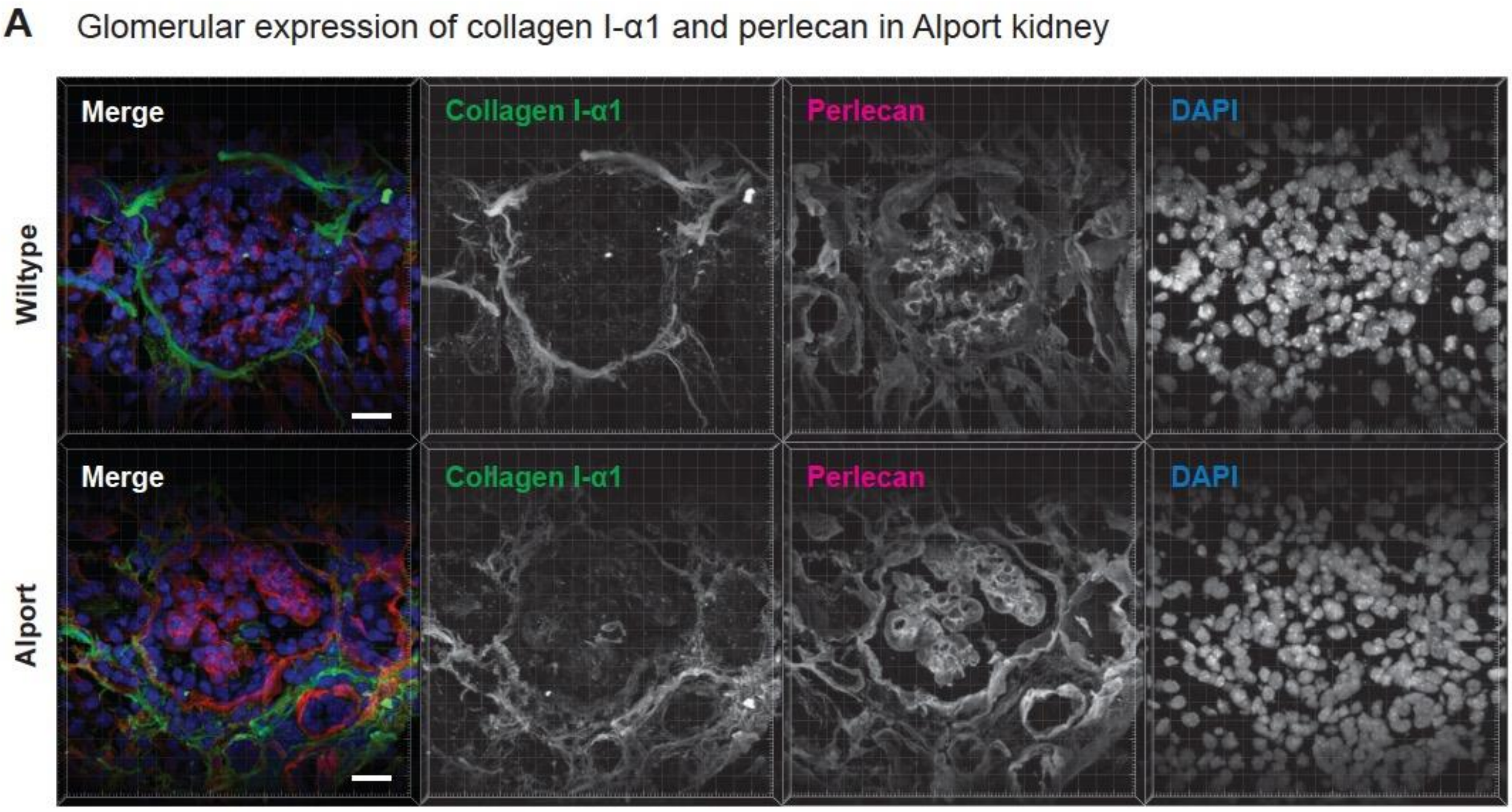

Supplementary Figure 6: Collagen I-α1 and perlecan expression in Alport kidney. A) Double-labelling immunofluorescence of COL1A1 (green) and GBM marker perlecan (magenta) in wildtype (upper panel) and Alport (lower panel) mice demonstrating accurate identification of glomeruli within kidney tissue sections and increased periglomerular COL1A1 expression in Alport kidney. Images acquired at 50X magnification with a Zeiss CD7 microscope. Bars, 15μm. N=3 biological replicates and n=3 technical replicates.

Supplementary Figure 7

A Principle components analysis of heavy-to-light ratios

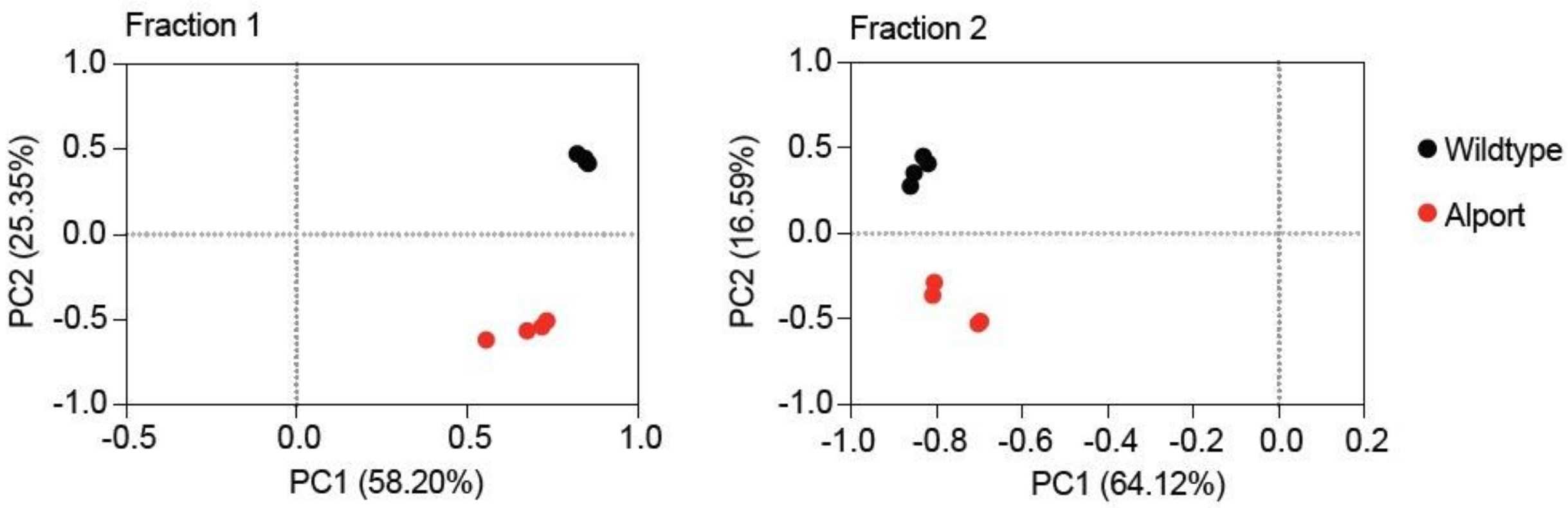

B Increased ^{13}C-Lysine incoorporation with pulse-time

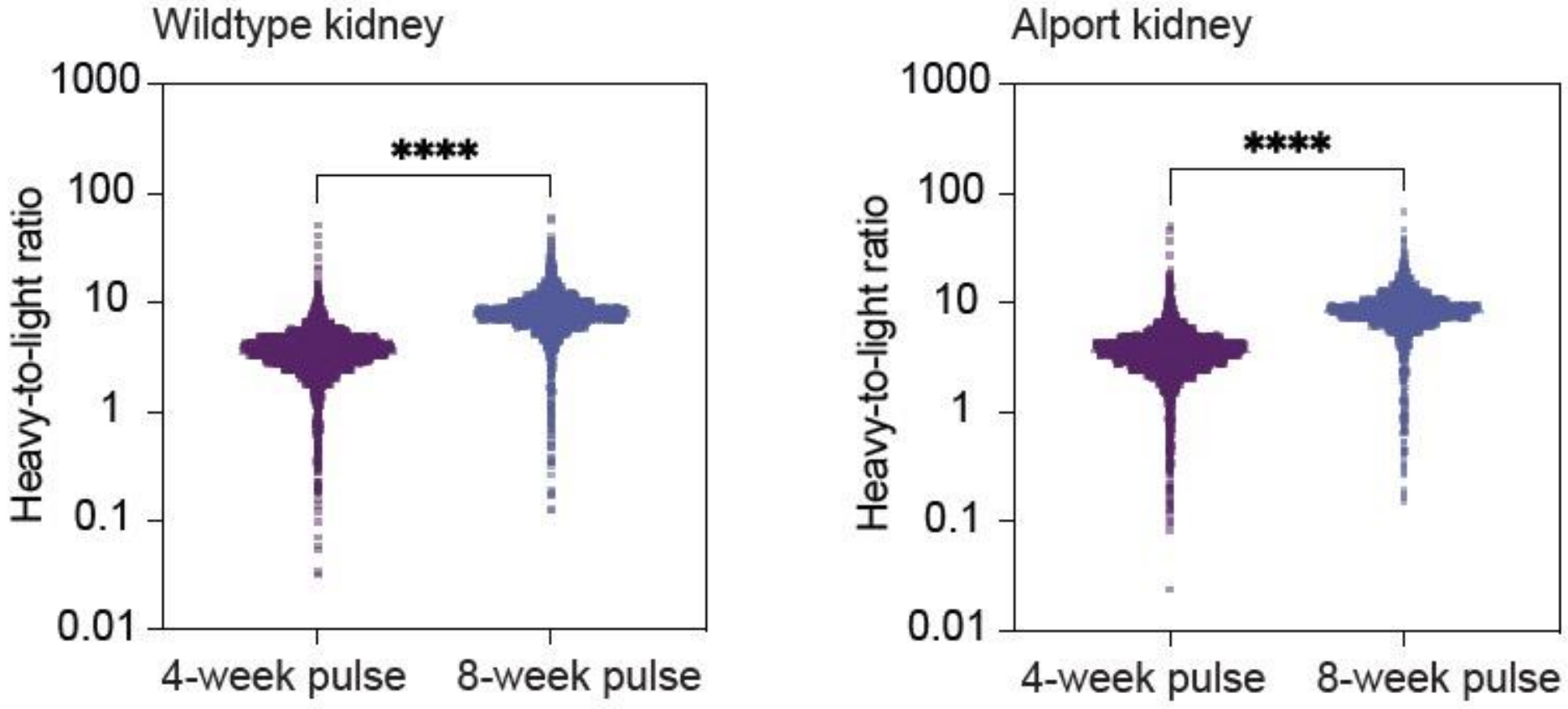

C Integrated analysis of transcriptomic and proteomic data

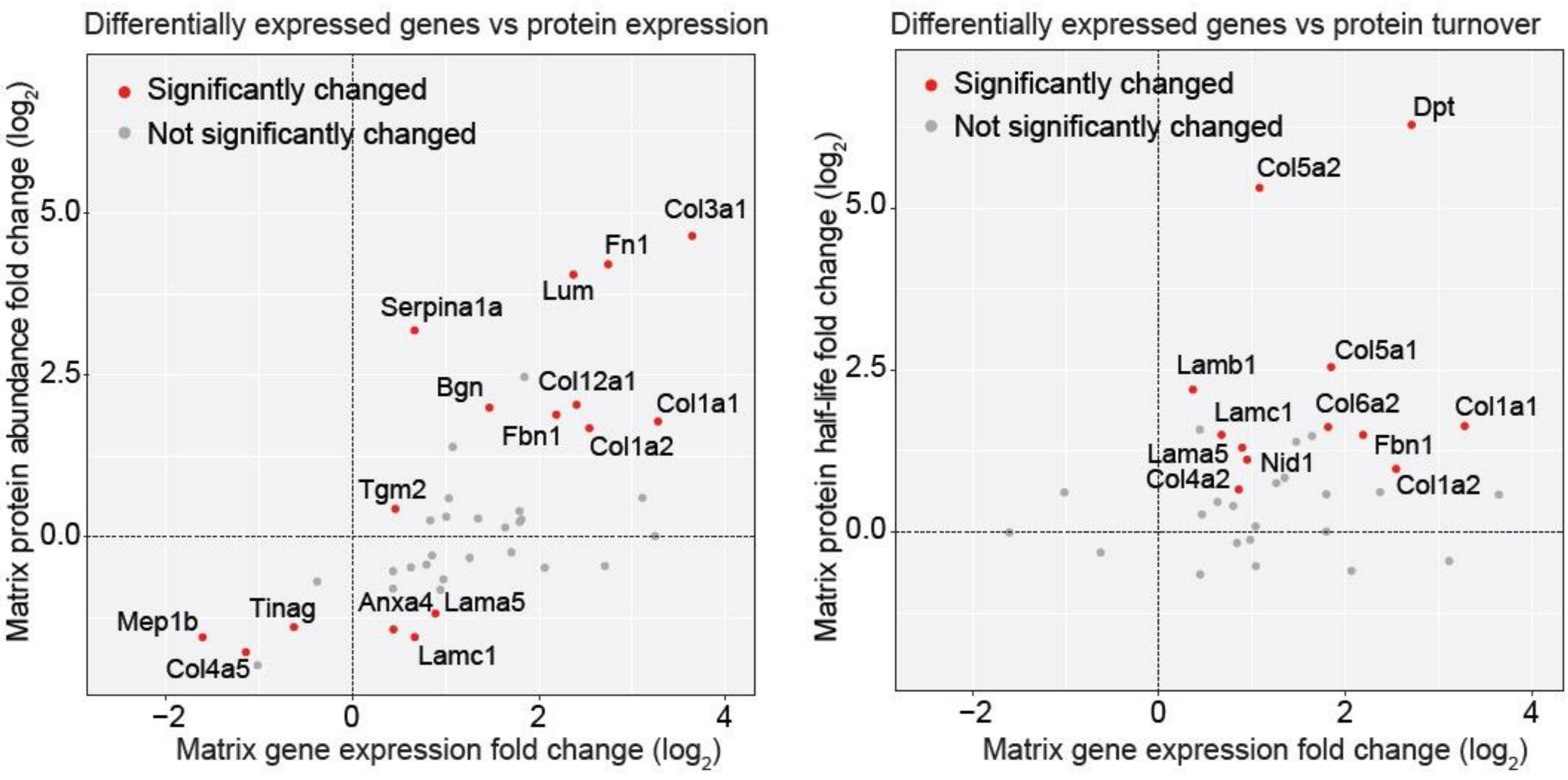

Supplementary Figure 7: Kidney matrix turnover in Alport kidney. A) Principal Components Analysis (PCA) of heavy-to-light ratios in Fraction 1 samples (left panel) and Fraction 2 samples (right panel) from wildtype (black dots) and Alport (red dots) kidney. **B**) Heavy-to-light ratios for all proteins detected in kidney tissue from wildtype (left panel) and Alport (right panel) mice fed ^{13}C-Lys diet for 4- or 8-week pulses showing increased ^{13}C-Lys incorporation over time. ****p=<0.0001 by Kruskal-Wallis test with correction for multiple comparisons. N=4 biological replicates per condition for all analysis. **C**) Integrated analysis of bulk RNA sequencing and proteomic data from wildtype and Alport kidney comparing fold change in matrix gene expression (x axis) with fold change in protein (y axis) expression (left panel) or half-life (right panel). Significantly changing genes and proteins are shown with red dots (adjusted p value < 0.05) and non-significantly changing genes and proteins are shown with grey dots. N=3 mice per condition for RNA sequencing and n=4 mice per condition for proteomics.

Supplementary Figure 8

A Matrix proteins with significant structural differences in Alport kidney

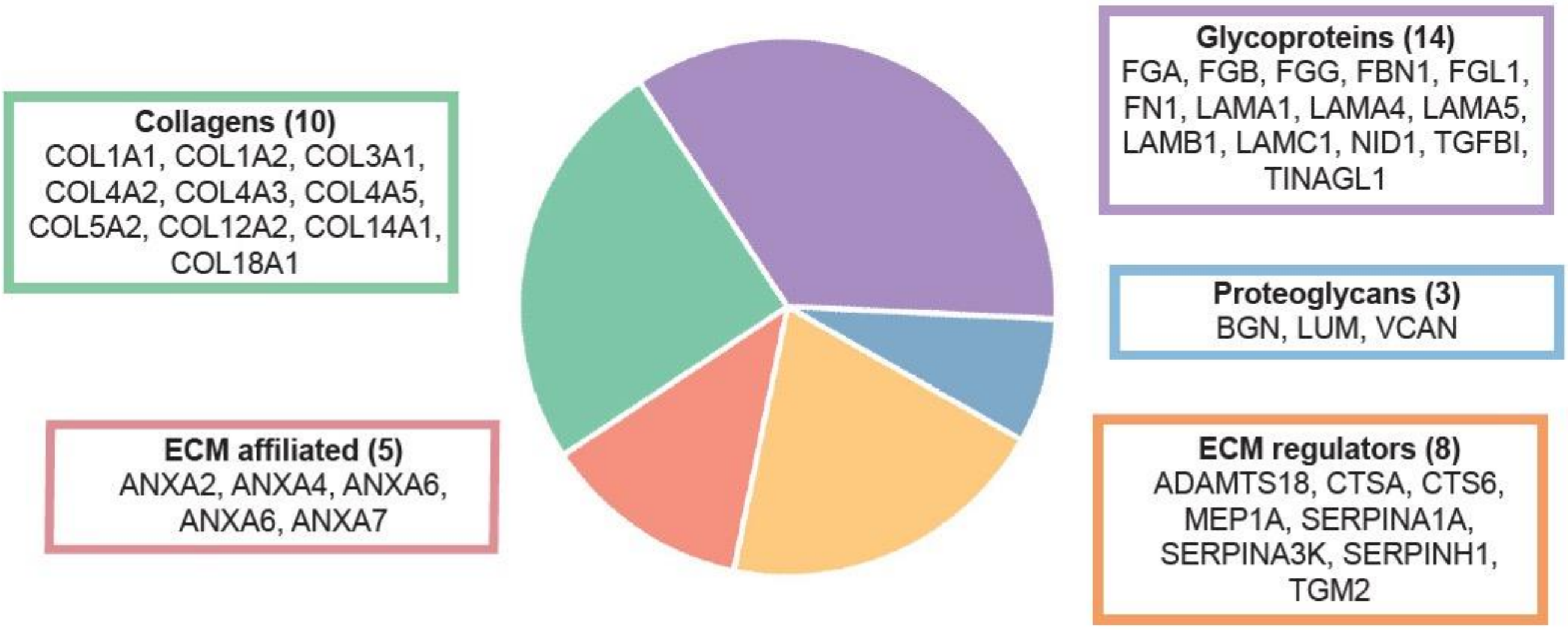

B Study participant characteristics

	**Alport cohort N=38**	**Healthy children N=30**
**Gender**		
Male	24 (63%)	19 (63%)
Female	14 (37%)	11 (37%)
**Age (years)**		
Range (median)	3 -18 (11.5)	9 – 15 (13)
**Ethnicity**		
White	19 (50%)	21 (70%)
Black	0 (0%)	4 (13.3%)
Asian	7 (18%)	1 (3.4%)
Not disclosed	12 (32%)	4 (13.3%)
**Genotype**		
X-linked (hemizygous)	12 (32%)	-
X-linked (heterozygous)	6 (16%)	-
Autosomal dominant (heterozygous)	18 (47%)	-
Autosomal recessive (homozygous)	2 (5%)	-
**Proteinuria**		
uPCR < 30 mg/mmol	23 (61%)	-
uPCR > 30 mg/mmol	15 (39%)	-
**Estimated GFR**		
< 90 mL/min/1.73m^2	3 (8%)	-
> 90 mL/min/1.73m^2	35 (92%)	-

Supplementary Figure 8: Detection of circulating matrix in Alport syndrome. A) A total of 40 matrix proteins were identified with statistically significant structure-associated differences between wildtype and Alport kidney. Matrix proteins with significant structural differences in Alport kidney were classified according to MatrisomeDB and grouped into collagens, glycoproteins, proteoglycans, ECM regulators and ECM-affiliated proteins. N=4 biological replicates per condition. **B**) Summary of cohort characteristics from children with Alport gene variants (n=38) and healthy children (n=30) used for detection of circulating matrix.

Supplementary Figure 9

A Increased endogenous collagenase activity in Alport collagen I

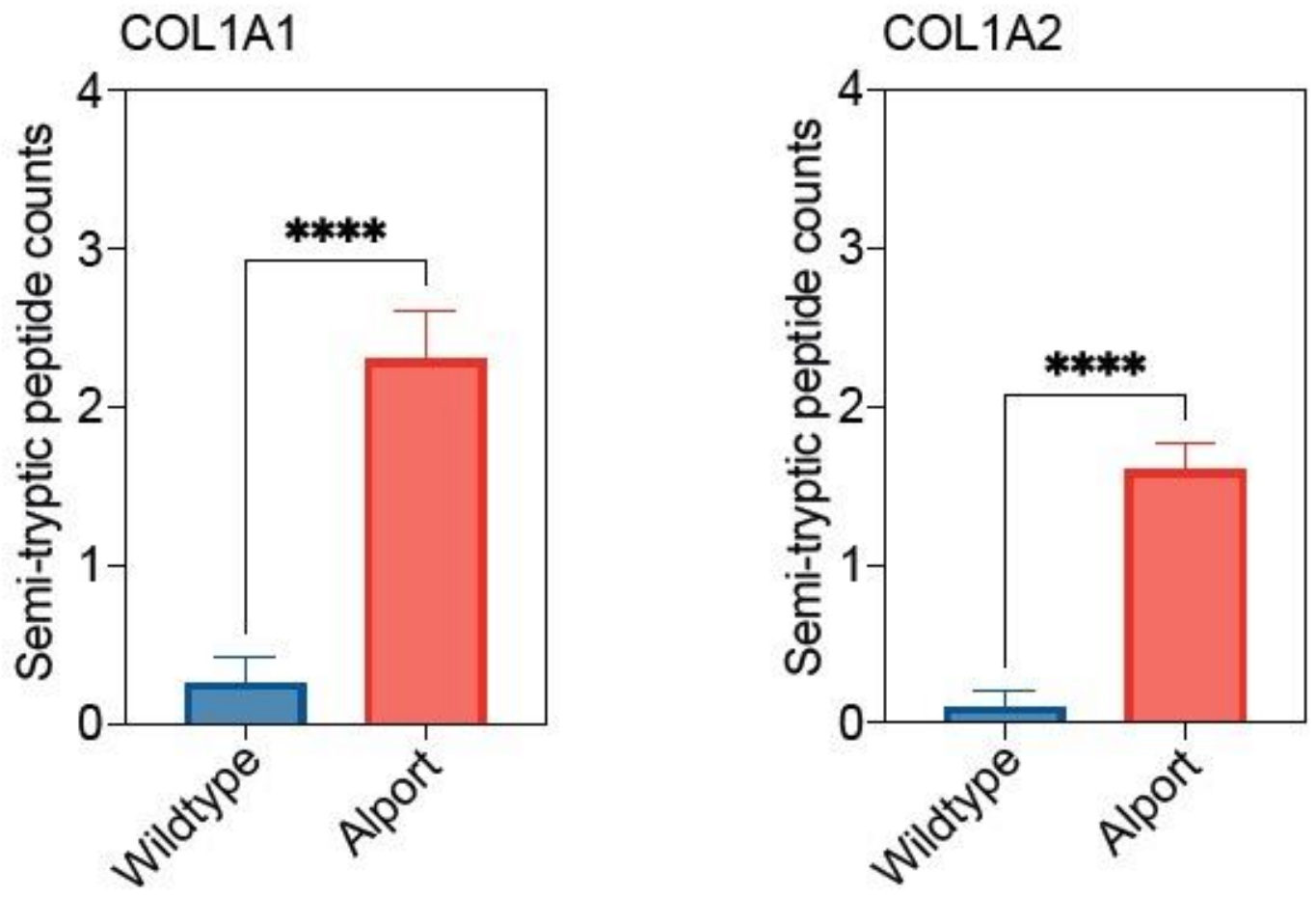

B Increased *in situ* collagenase activity in Alport glomeruli

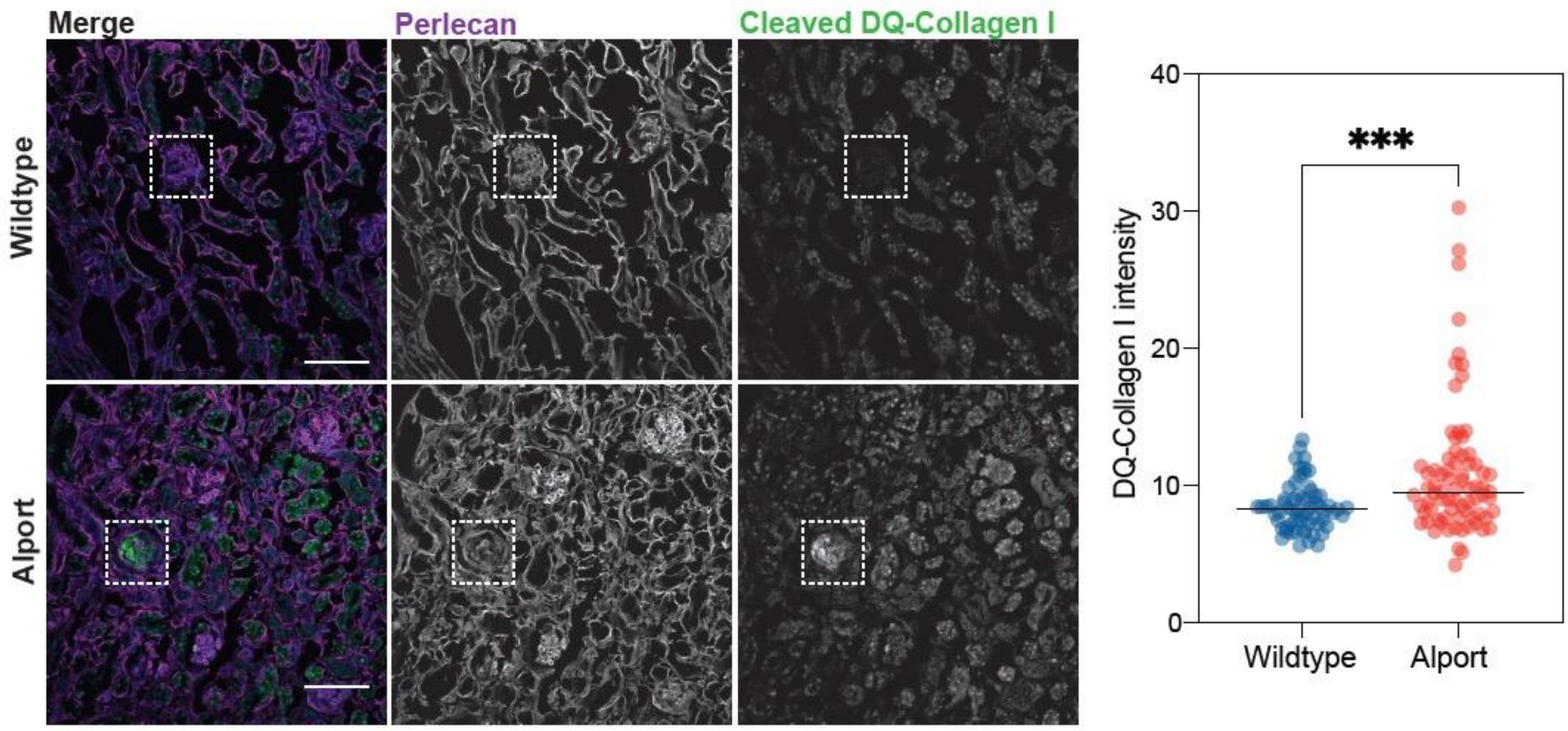

C Increased circulating MMP-mediated collagen fragments in Alport mice

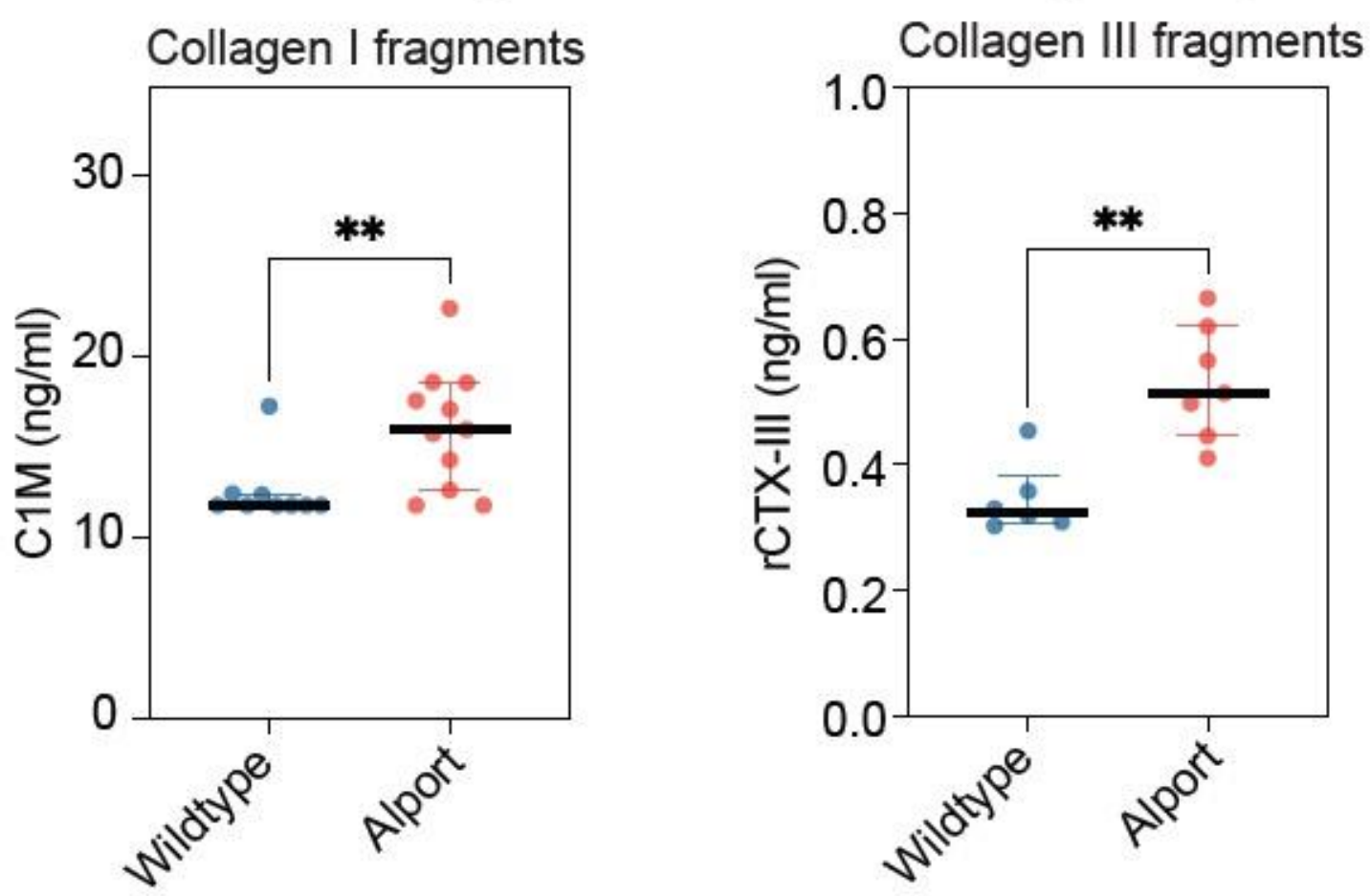

Supplementary Figure 9: Increased protease activity in Alport kidney. A) Total counts of COL1A1- (left panel) and COL1A2-derived (right panel) semi-tryptic peptides in wildtype and Alport mice. ****p= <0.0001 by unpaired t-test. Bars show mean±SEM. **B**) Dual immunofluorescence staining for perlecan (purple) and cleaved DQ-Collagen I in wildtype (upper panel) and Alport (lower panel) kidney. Left panel: Representative glomeruli highlighted by dashed box showing increased DQ-Collagen I signal in Alport kidney, indicating increased *in situ* collagenase activity. Images acquired at 20X magnification with a Leica SP8 confocal microscope. Bars, 100 μm. N=5 biological replicates per condition. Right panel: Quantification of 63 glomeruli (10, 6, 13, 23, 11) and 80 glomeruli (20, 11, 14, 15, 20) randomly selected from wildtype and Alport kidney sections. ***p=0.0003 by Mann Whitney test. Bars represent median with 95% confidence intervals. **C**) Analysis of mouse serum (C1M) or plasma (rCTX-III) from wildtype and Alport mice demonstrating significantly increased levels of circulating MMP-mediated collagen I fragments (left panel) and MMP-mediated collagen III fragments in Alport mice. **p=0.0094, **p=0.0047 by Mann Whitney test. Bars represent median with 95% confidence intervals. For serum analysis (C1M), n=9 wildtype and n=11 Alport mice. For plasma analysis (rCTX-III), n=6 wildtype and n=7 Alport mice.

Supplementary Figure 10

A COL1A1

i [R].GFPGADGVAGPKGPSGER.[G], position 483-500

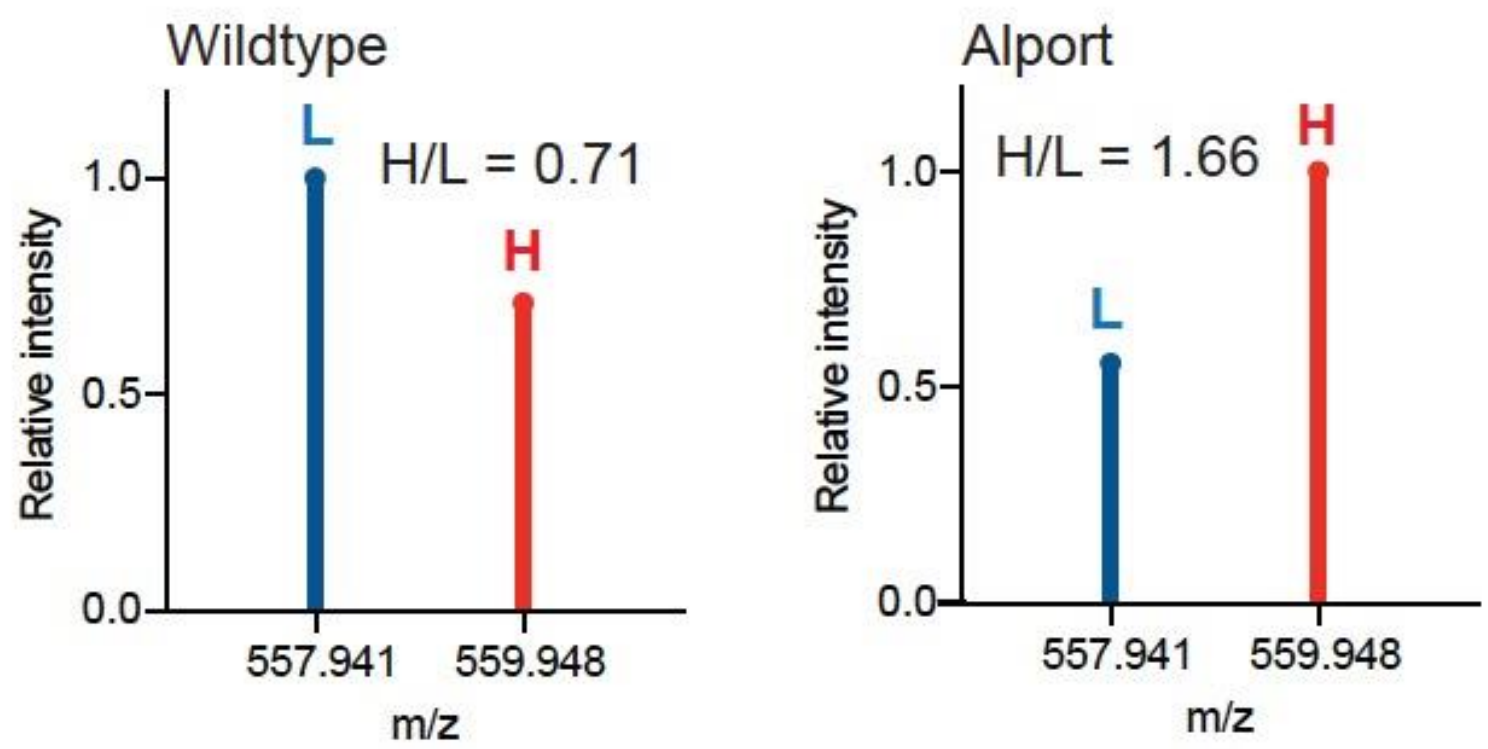

ii [R].GLTGPIGPPGPAGAPGDKGEAGPSGPPGPTGAR.[G], position 753-785

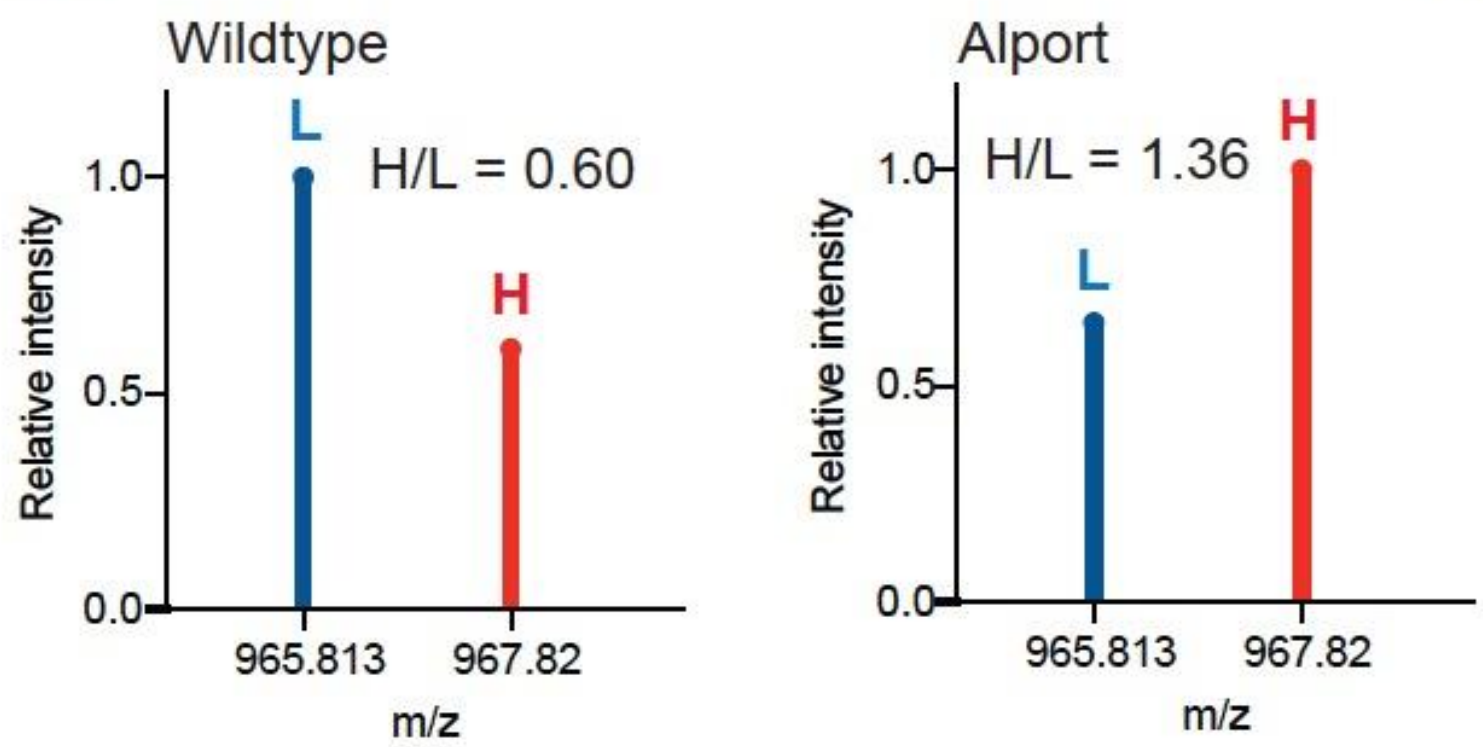

B LAMA5

i [R].STDFGHTYQPWQFFASSKR.[D], position 180-198

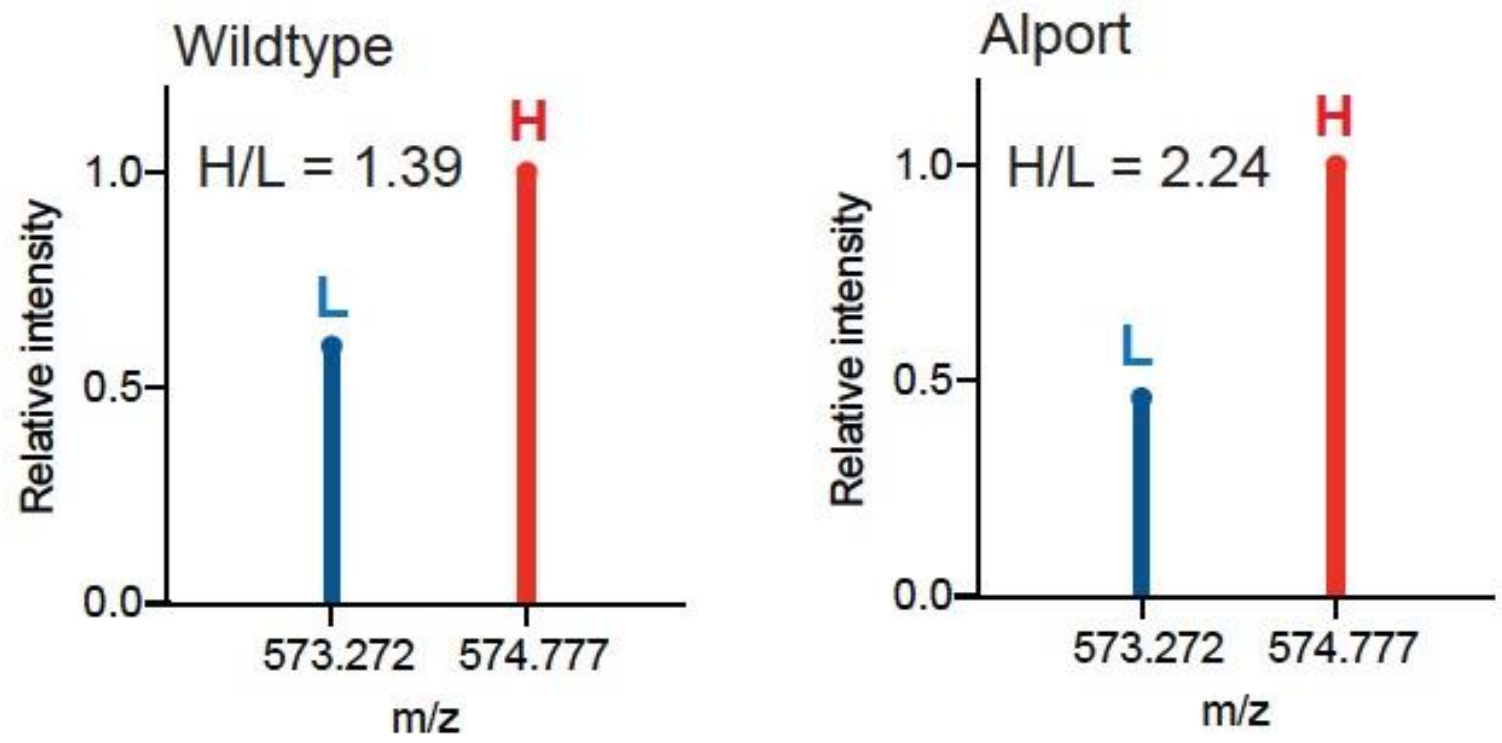

ii [R].DASSSVSTLEK.[T], position 2680-2690

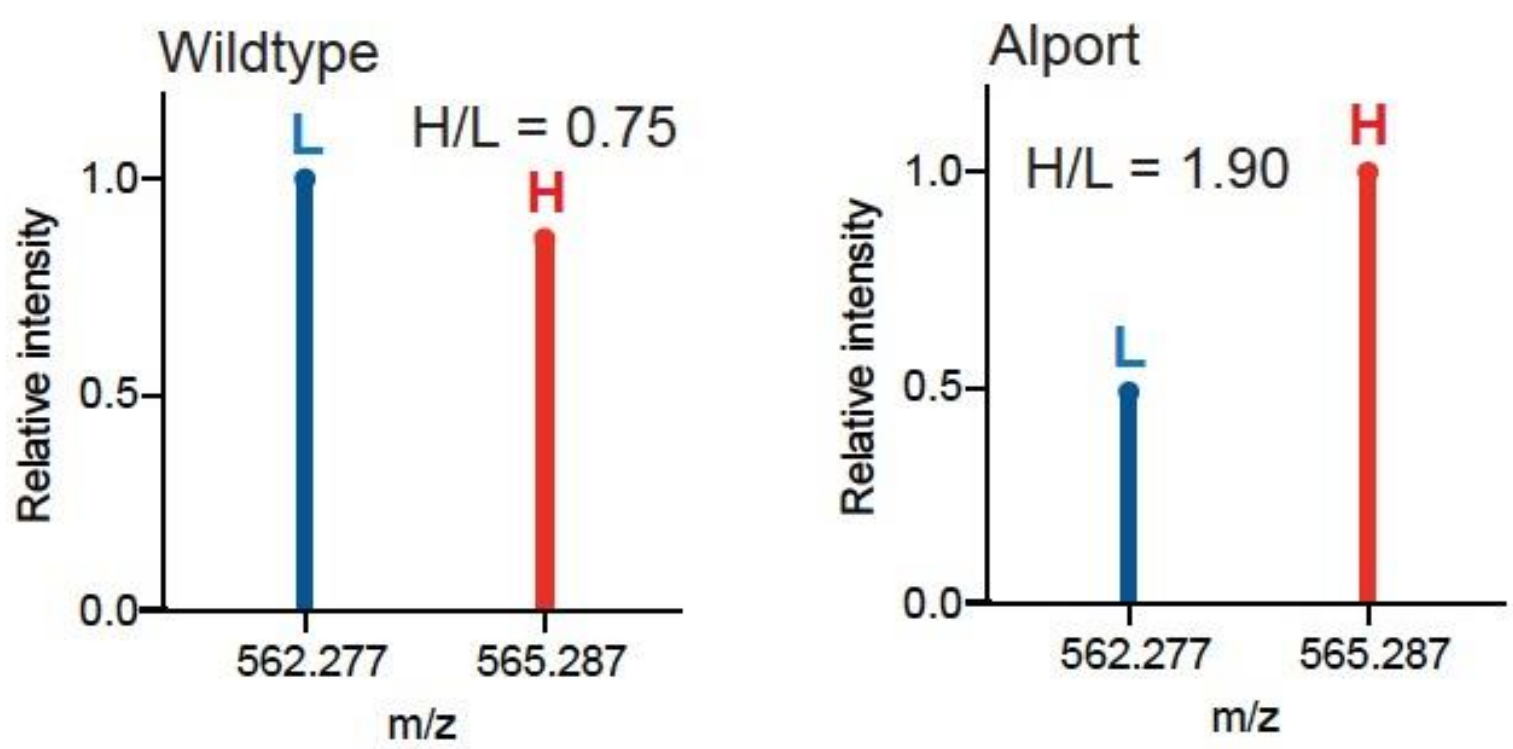

Supplementary Figure 10: Representative MS1 spectra illustrating variation in ^{13}C-lysine incorporation across protein sequences. A) Representative MS1 precursor ion spectra for two tryptic peptides from COL1A1 detected in wildtype (left panel) and Alport (right panel) kidney. **i)** Peptide [R].GFPGADGVAGPKGPSGER.[G] (position 483-500), located outside the region of structural interest identified by peptide location fingerprinting analysis (GXY structure, 750-800 AA). **ii)** Peptide[R].GLTGPIGPPGPAGAPGDKGEAGP SGPPGPTGAR.[G] (position 753-785), located within the region of structural interest. **B)** Representative MS1 precursor ion spectra for two tryptic peptides from LAMA5 detected in wildtype (left panel) and Alport (right panel) kidney. **i)** Peptide [R].STDFGHTYQPWQFFA SSKR.[D] (position 180-198), located outside the region of structural interest (coiled-coil domain, 2600-2700 AA). **ii)** Peptide [R].DASSSVSTLEK.[T] (position 2680-2690), located within the region of structural interest. For each spectrum, blue bars represent light (L, pre-existing protein) and red bars represent heavy (H, newly synthesised ^{13}C-lysine labelled protein) precursor ions. Relative intensity is normalised to the tallest peak. Heavy-to-light (H/L) ratios represent the median of n=4 biological replicates (Alport and wildtype) with the exception of DASSSVSTLEK where n=3 biological replicates were available.
